# A genetic screen in *Drosophila* reveals an unexpected role for the KIP1 ubiquitination-promoting complex in male fertility

**DOI:** 10.1371/journal.pgen.1009217

**Published:** 2020-12-30

**Authors:** Weizhe Li, Jinqing Liang, Patricia Outeda, Stacey Turner, Barbara T. Wakimoto, Terry Watnick

**Affiliations:** 1 Division of Nephrology, Department of Medicine, University of Maryland School of Medicine, Baltimore, MD, United States of America; 2 Department of Biology, University of Washington Seattle, WA, United States of America; Washington University School of Medicine, UNITED STATES

## Abstract

A unifying feature of polycystin-2 channels is their localization to both primary and motile cilia/flagella. In *Drosophila melanogaster*, the fly polycystin-2 homologue, Amo, is an ER protein early in sperm development but the protein must ultimately cluster at the flagellar tip in mature sperm to be fully functional. Male flies lacking appropriate Amo localization are sterile due to abnormal sperm motility and failure of sperm storage. We performed a forward genetic screen to identify additional proteins that mediate ciliary trafficking of Amo. Here we report that Drosophila homologues of KPC1 and KPC2, which comprise the mammalian KIP1 ubiquitination-promoting complex (KPC), form a conserved unit that is required for the sperm tail tip localization of Amo. Male flies lacking either KPC1 or KPC2 phenocopy *amo* mutants and are sterile due to a failure of sperm storage. KPC is a heterodimer composed of KPC1, an E3 ligase, and KPC2 (or UBAC1), an adaptor protein. Like their mammalian counterparts Drosophila KPC1 and KPC2 physically interact and they stabilize one another at the protein level. In flies, KPC2 is monoubiquitinated and phosphorylated and this modified form of the protein is located in mature sperm. Neither KPC1 nor KPC2 directly interact with Amo but they are detected in proximity to Amo at the tip of the sperm flagellum. In summary we have identified a new complex that is involved in male fertility in *Drosophila melanogaster*.

## Author summary

In *Drosophila*, the polycystin-2 homologue, Amo, is found in a restricted area at the tip of the sperm tail where it mediates directed sperm migration and proper sperm storage. The factors involved in Amo trafficking to this distinctive subcellular domain are unknown. We screened a collection of male sterile *Drosophila* EMS mutants for mislocalization of Amo to gain clues about what proteins that might be involved in this process. Here we report that fly homologues of KPC1 and KPC2 which, form the core of the mammalian KIP1 ubiquitination-promoting complex (KPC) are required for Amo to reach the tip of the sperm tail. In summary, we have uncovered a novel role for the KIP1 ubiquitination-promoting complex in Drosophila sperm and male fertility.

## Introduction

TRPP2 (polycystin-2, PC2) proteins are non-selective, calcium permeable channels that are evolutionarily conserved from algae to humans [1]. In humans, mutations in *TRPP2* (*PKD2*) result in autosomal dominant polycystic kidney disease (ADPKD), which is the most common monogenic cause of renal failure [2,3]. Polycystin-1 (PC1), the product of the *PKD1* gene, serves as the primary binding partner of TRPP2 and mutations in this gene also result in ADPKD [2,4]. A unifying feature of TRPP2 channels and their polycystin-1 receptors is their conserved localization to both primary and motile cilia/flagella where they are thought to participate in signaling cascades that regulate diverse processes including male mating behavior, renal tubular morphogenesis and left/right axis determination [5–10].

We have previously demonstrated that in *Drosophila melanogaster*, theTRPP2 homologue, Amo, has a distinctive localization at the tip of the sperm flagellum [11,12]. Males lacking Amo are sterile due to a failure of sperm to reach specialized female storage organs, the seminal receptacle and spermathecae [11]. Amo regulates flagellar beating patterns, which are required for directed sperm motility and ultimately for male fertility [12]. Although there is a large pool of Amo located in the endoplasmic reticulum (ER) of developing spermatocytes, the protein must ultimately cluster at the flagellar tip in mature sperm to be fully functional. The factors that are responsible for Amo trafficking to this membrane domain are unknown.

Cilia and flagella are evolutionarily conserved microtubule based organelles that form an antenna like projection emanating from the surface of most cell types [13]. The ciliary membrane is continuous with the apical plasma membrane and houses a distinct set of receptors that are involved in signaling cascades that are regulated at the cilium [14]. Since no protein synthesis occurs in cilia/flagella, the mechanisms responsible for trafficking of integral membrane proteins to the cilium remains a subject of intense investigation [15,16].

In the case of the vertebrate polycystin complex both PC1 and PC2 have been reported to harbor ciliary targeting sequences but these motifs appear to be insufficient for sorting polycystins to the cilium [17–20]. A series of elegant studies suggest that a direct interaction between PC1 and TRPP2 in the endoplasmic reticulum and then PC1 cleavage at its GPS site are critical steps in moving the complex to the trans-Golgi on the way to the cilium [20–22]. This route through the Golgi suggests that the secretory pathway and vesicular transport machinery is likely to be involved in the ciliary targeting of the polycystin complex. Indeed, independent yeast two hybrid screens using the C-terminus of PC1 have implicated GGA1 and its adaptors as well as the BBSome in ciliary trafficking of the PC1/PC2 complex in cultured cells [20,23]. In addition, TRPP2 has been shown to bind to the exocyst protein Sec 10 in HEK cells [24]. The exocyst is a conserved octameric complex that is thought to regulate primary ciliogenesis by mediating the tethering of secretory vesicles carrying membrane proteins to the base of the cilium [25]. Morpholino knock down of Sec 10 in zebrafish results in a subset of phenotypes that is also associated with *Pkd2* deficiency [24]. However, conditional deletion of Sec10 in the murine kidney using a Ksp-Cre recombinase results in ureteropelvic junction obstruction rather than polycystic renal disease [26]. How the various protein complexes are integrated with respect to polycystin trafficking *in vivo* remains unknown.

In the present study, we took advantage of genetic tools available in flies and performed an unbiased screen to identify additional factors that mediate ciliary trafficking of *Drosophila* TRPP2, Amo. We screened a collection of male sterile mutants for mislocalization of Amo and found that components of the *Drosophila* KIP1 ubiquitination-promoting complex (KPC) are required for the sperm tip localization of Amo. KPC is a heterodimer composed of KPC1, an E3 ligase, and KPC2 (or UBAC1), an adaptor protein [27–29]. We found that KPC1 and KPC2 don’t directly interact with Amo, but they are detected in proximity to Amo at the tip of the sperm flagellum. We conclude that the KPC complex is necessary for male fertility in Drosophila by ensuring that Amo reaches a membrane domain at the tip of the sperm flagellum, where it is required for proper sperm storage.

## Results

### A screen of male sterile mutants identifies a novel sperm storage mutant with defective dPKD2 sperm localization

**Fig 1:**
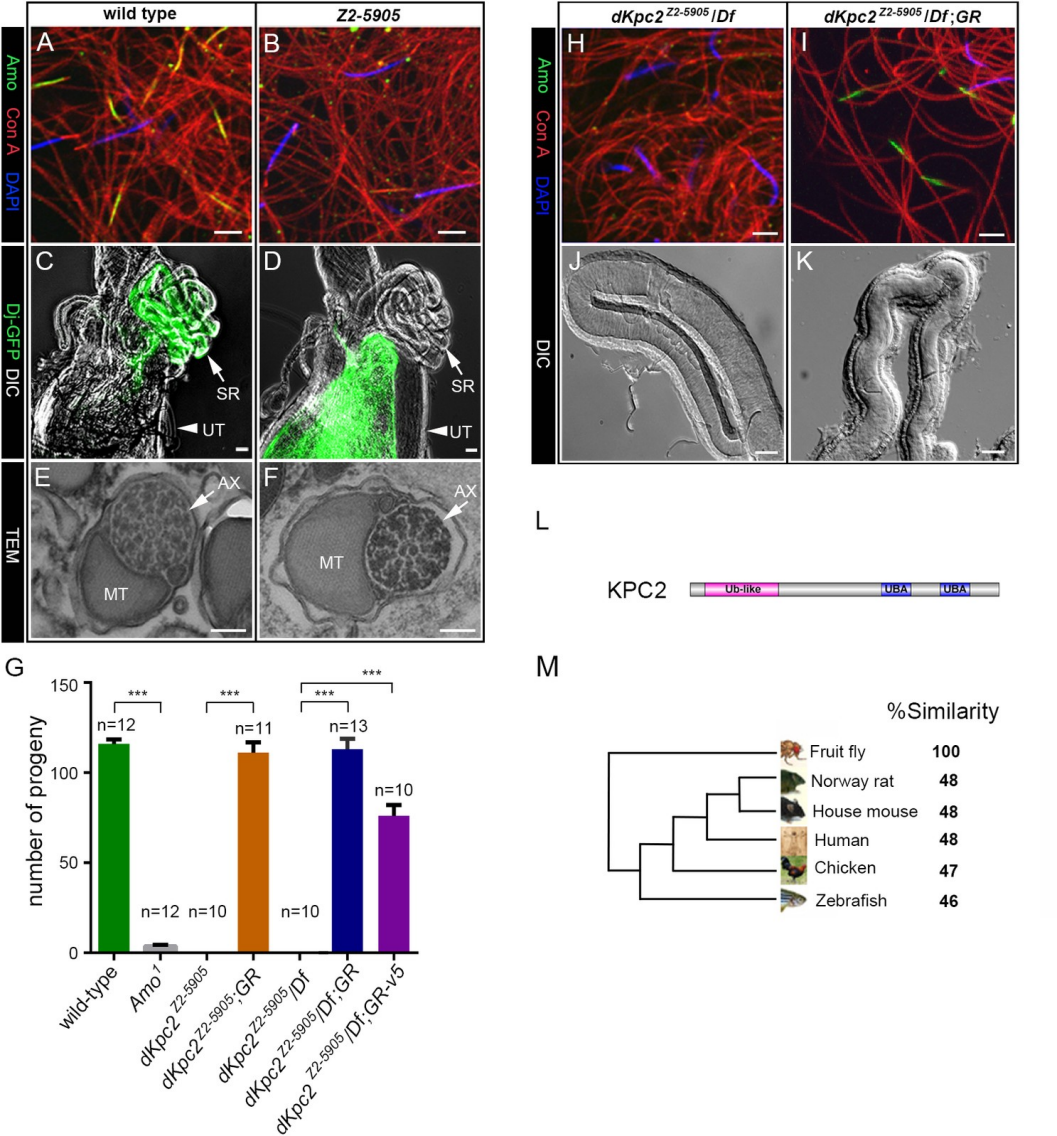
The *Drosophila* homolog of KPC2 is required for sperm tail localization of AMO. (A-B) Sperm stained with anti-Amo: green, concanavalin A: red, DAPI: blue. Amo is missing from the sperm tail in Z2-5905 sperm (B). Scale bars: 5 μm. (C-D) Wild type female reproductive tracts dissected after mating with *dj-*GFP males (C) or *Z2-5905; dj-*GFP males (D). DIC and fluorescence images are super-imposed. Wild type sperm are found in the seminal receptacle (SR) while Z2-5905 mutant sperm are restricted to the uterus (UT). Scale bars: 100 μm. (E-F) Representative electron microscopy images from wild type (E) and Z2-5905 (F) sperm. Scale bars: 100 nm. MT: mitochondria AX: axoneme. (G) Fertility tests with males of the indicated genotypes mated to wild type females. The male fertility defect in Z2-5905 mutants is rescued by a CG11025 genomic rescue (GR) construct with or without a V5 tag. The number of tests per genotype is denoted above the bars. Df: deficiency. ***p<0.001. (H-I) Sperm stained with anti-Amo: green, concanavalin A: red, DAPI: blue. Scale bars: 5 μm. (J-K) Representative DIC images of wild type seminal receptacles dissected after mating with Z2-5905 males (J) or Z2-5905 males with a genomic rescue (K). The genomic construct rescues both Amo localization and sperm storage. Scale bars: 20 μm. (L) Predicted domain structure of all three KPC2 isoforms showing an N-terminal ubiquitin like domain (Ub-like) and two C-terminal ubiquitin associated domains (UBA). (M) Phylogenetic tree demonstrates homology between *Drosophila* KPC2 and related proteins.

To identify new proteins that might be involved in AMO trafficking, we screened a subset of 60 male sterile EMS mutants producing motile sperm, which were isolated from the Zuker Collection [30,31]. We reasoned that mis-localization of *Drosophila* PKD2 (AMO) at the tip of the sperm tail should phenocopy the *Amo* null mutant and result in deficient sperm storage [11]. We identified a mutant line on chromosome 2, Z2-5905, that lacked Amo staining at the tip of the sperm flagellum **(Fig 1A and 1B)** [11, 12]. Mutant Z2-5905 testes, however, showed a wild type pattern of Amo staining in earlier stages of sperm maturation **(S1A–S1D Fig)**. In particular Amo exhibited the previously reported ER localization pattern in primary spermatocytes **(S1I–S1N Fig)** [12]. Detailed phenotypic analysis revealed that Z2-5905 mutant males court and mate normally but sperm from these males failed to reach either the seminal receptacle or the spermathecae in single pair matings with wild type females **(Fig 1C and 1D and S1E and S1F Fig).**

We further characterized spermatogenesis in Z2-5905 mutant testes by introducing *protB-DsRed* and *dj-GFP* transgenes, which mark sperm heads and tails, respectively **(S1G and S1H Fig)**. We found that sperm development and individualization proceeded normally in Z2-5905 mutants. In addition, electron microscopy did not detect any gross abnormalities in the ultrastructure of Z2-5905 sperm tails **(Fig 1E and 1F).**

### The gene mutated in Z2-5905 encodes a *Drosophila melanogaster* homologue of UBAC1/dKPC2

To identify the causal mutation in Z2-5905, we used a combination of meiotic mapping, deficiency mapping and exon sequencing and found a splice site mutation in *CG11025* **(S2A and S2B Fig).** *CG11025* is predicted to encode three transcripts: A (2479 base pairs, 557 amino acids), B (1778 base pairs, 435 amino acids) and C (2491 base pairs, 561 amino acids) **(S2A Fig).** The mutation affects the splice donor site of a small coding exon contained in transcripts B and C **(S2A and S2B Fig)**. Reverse-Transcription Polymerase Chain Reaction (RT-PCR) analysis using transcript specific primers showed that only transcripts B and C are expressed in wild type flies, and transcript B is testis-specific **(S2C–S2E Fig and S1 Table)**. RT-PCR and sequencing of transcript B from Z2-5905 testes confirmed that the splice site mutation causes inclusion of the intron between exons 1 and 2, which is predicted to result in a novel protein and a premature stop codon after 61 amino acids. Transcript C was not detected in Z2-5905 flies, presumably due to nonsense mediated decay **(S2D Fig)**. We rescued male fertility, sperm storage and Amo localization in Z2-5905 flies by transgenic re-expression of a CG11025 genomic construct containing native promoter elements **(Fig 1G–1K** and **S1 Data)**. This confirms that the splice site mutation in CG11025 is responsible for the male sterile phenotype of Z2-5905 flies.

We analyzed the predicted protein sequence of Transcripts B and C, which differ only at their amino termini, with Isoform C being 126 amino acids longer. Both transcripts encode polypeptides with two C-terminal ubiquitin associated (UBA) domains and an N-terminal ubiquitin-like domain **(Fig 1L)**. Blast analysis suggested that CG11025 encodes the fly homologue of a vertebrate gene, *UBAC1*, also known as *KPC2*. Isoform B is 30% identical and 48% similar to human KPC2 (E value 2e^-35^) **(Fig 1M)**. KPC2 is an adaptor that forms part of the KIP1 ubiquitin-promoting complex [27]. KPC2 interacts with ubiquitinated proteins and the proteasome and promotes the degradation of the cyclin dependent kinase inhibitor p27Kip1[27–29]. For simplicity, we will refer to CG11025 as *dKPC2*.

### Generation of a *dKpc2* Null Allele

**Fig 2:**
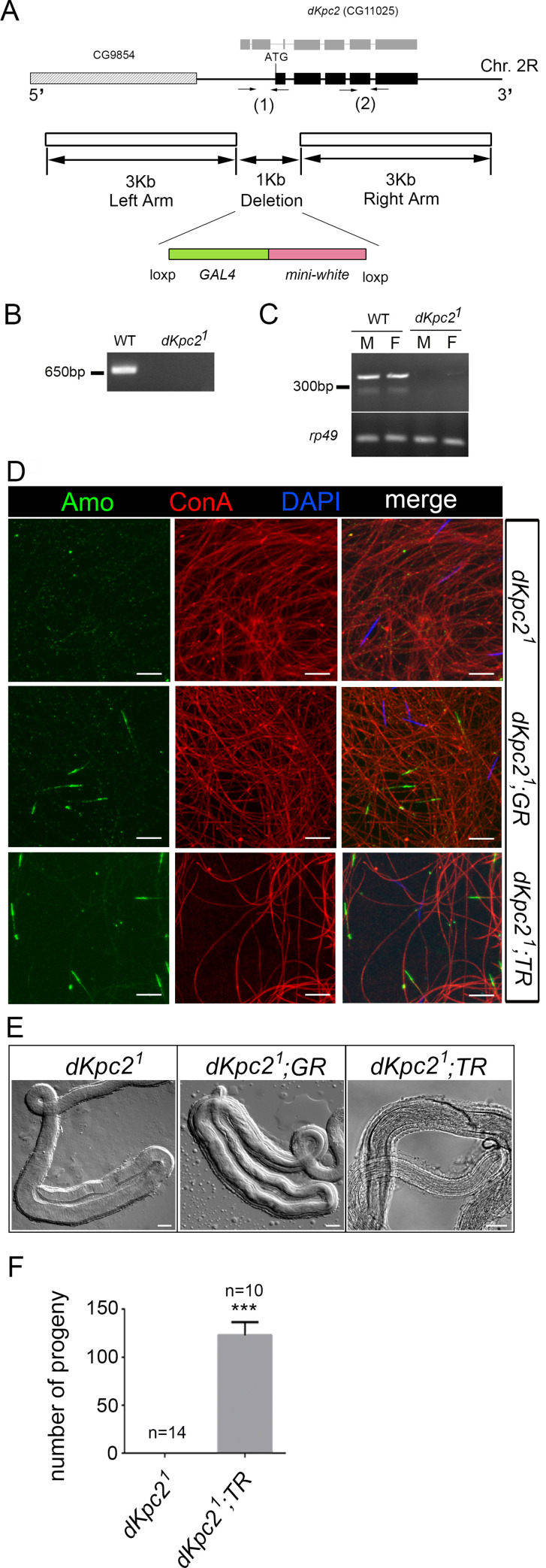
Generation of a *dKpc2* mutant allele (*dKpc2*^*1*^) by homologous recombination. (A) Physical map of the *dKpc2 (CG11025)* locus and the targeting scheme used to generate the *dKpc2*^*1*^ allele by homologous recombination. The solid boxes represent exons in Transcript B (black) and in Transcript C (gray). Arrows indicate the location of PCR primers. (B) Genomic PCR using primer pair (1) confirms a ~1KB deletion at the 5’ end of *dKpc2*. (C) RT-PCR with primer pair (2) confirms the absence of a *dKpc2* transcript in mutant males (M) and Females (F). *rp49* is a housekeeping gene. (D) Sperm stained with anti-Amo: green, concanavalin A: red and DAPI: blue. Amo is missing from *dKpc2*^*1*^ sperm tails but localization is rescued by a CG11025 genomic construct (*dKpc2*^*1*^; *GR*) and by testis specific expression of *dKpc2*, Transcript B (*dKpc2*^*1*^; *TR*). Scale bars: 10 μm. (E) DIC images of Seminal receptacles (SR) from wild type females after mating with males of indicated genotypes. The SR is empty after mating with *dKpc2*^*1*^males. Sperm storage is rescued by the CG11025 genomic construct (*dKpc2*^*1*^; *GR*) and by testis-specific expression of Transcript B (*dKpc2*^*1*^; *TR*). Scale bars: 20 μm. (F) Fertility tests with males of the indicated genotypes mated to wild type females. The number of tests per genotype is denoted above the bars. ***p< 0.001.

We used ends-out homologous recombination to generate a second mutant allele of CG11025, *dKpc2*^*1*^ **(Fig 2A–2C)**. We replaced the N-terminal coding region of *dKpc2* with Gal4 coding sequences and deleted the predicted initiation codon of both transcripts B and C. *dKpc2*^*1*^ mutant males exhibited the same phenotype as the EMS mutant, Z2-5905 (**Fig 2D–2F** and **S1 Data**). The males were viable but sterile due to a sperm storage defect and Amo was missing at the tip of the sperm flagellum. These defects could be rescued by transgenic re-expression of the CG11025 genomic construct (**Fig 2D and 2E**). In addition, using the β2-tubulin promoter to direct testis-specific expression of transcript B was sufficient to fully rescue male fertility and all associated defects **(Fig 2D–2F** and **S1 Data)**. This suggests that the functional isoform of dKPC2 in the testis is encoded by isoform B.

### *d*KPC2 encodes a sperm specific protein that is monoubiquitinated and phosphorylated

**Fig 3:**
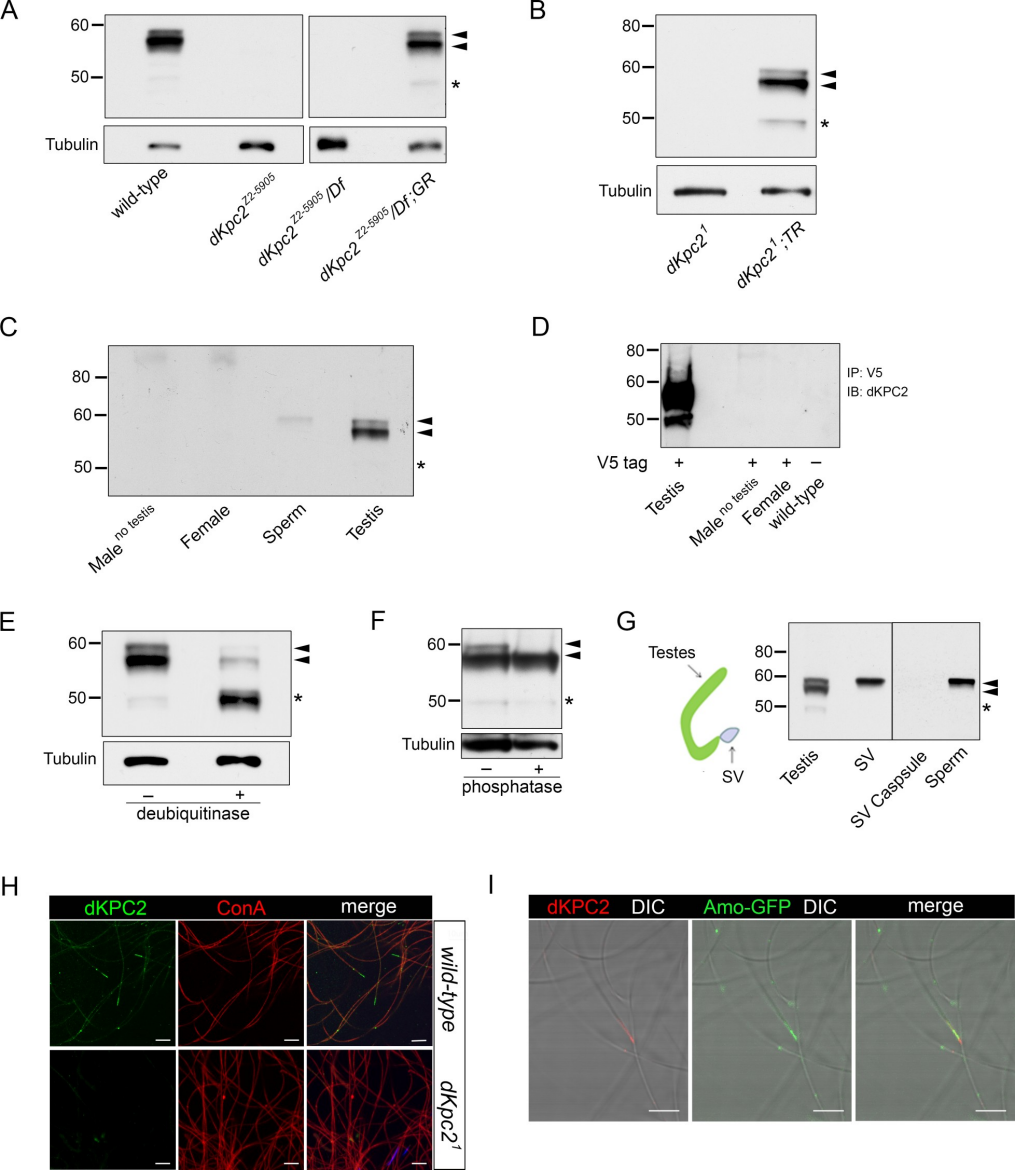
*Drosophila* KPC2 is a mono-ubiquitinated protein that co-localizes with Amo at the tip of the sperm tail. (A-G) Western blots probed with antisera to dKPC2. (A, B) Anti-dKPC2 recognizes three isoforms in wild type male testes indicated by arrowheads and an asterisk. These bands are absent in Z2-5905 (A) and *dKpc2*^*1*^ (B) mutant testes. All three dPKC2 isoforms are visualized upon re-expression of dKPC2 using a genomic rescue construct (GR) or by testis-specific re-expression of *dKpc2* Transcript B (*dKpc2*^*1*^; *TR*). Loading control: Tubulin. (C) dKPC2 is expressed in testis and sperm but cannot be detected in females or in males lacking testes. (D) Anti-V5 was used to immunoprecipitate (IP) lysates prepared from transgenic flies with a V5 tagged-*dKpc2* genomic construct. dKPC2 is expressed in testis but is absent in females and males lacking testes. (E-F) Lysates were prepared from wild type testes and treated with either deubiquitinase (E) or phosphatase (F). In (E), deubiquitinase shifts the ~58 kDa doublet down to ~49 kDa, suggesting that it is monoubiquitinated. In (F), phosphatase shifts the upper band in the ~58kDa doublet down suggesting that dKPC2 is monoubiquitinated and phosphorylated. Black arrow heads indicate the dKPC2 doublet at ~58kDa. The asterisk denotes the unmodified product of Transcript B at ~49kDa. (G) Schematic of the male reproductive tract showing structures that were dissected and used to prepare Western blots. Only the largest dKPC2 band is present in mature sperm. SV: seminal vesicle. (H) Wild type and *dKpc2*^*1*^ sperm stained with anti dKPC2: green, concanavalin A: red and DAPI: blue. dKPC2 localizes to the tip of the sperm tail. The antisera does not stain *dKpc2*^*1*^ sperm. Scale bars: 10 μm. (I) Amo-GFP sperm stained with antisera to dKPC2: red and to GFP: green. DIC and fluorescence images are merged showing that the two proteins partially co-localize at the tip of the sperm tail. Scale bars: 10 μm.

We raised polyclonal rabbit antibodies to dKPC2 that recognized a major ~58 kDa doublet on Western blots prepared from wild type males **(Fig 3A and S3A Fig)**. Close inspection revealed that the antibody also detected a minor dKPC2 species at ~49kDa, which is the predicted size of the polypeptide encoded by transcript B. All three bands were absent in *dKpc2* mutant flies but were detected upon transgenic re-expression of dKPC2 using the genomic rescue construct or by testis specific re-expression of Isoform B alone **(Fig 3A and 3B)**. dKPC2 was absent in lysates prepared from females and males where the testes had been dissected away **(Fig 3C)**.

To prove that all three bands observed on Western analysis were indeed related to dKPC2, we generated transgenic flies bearing a genomic *dKpc2* construct with a C-terminal V5 tag **(Fig 3D and S3B Fig)**. Immunoprecipitation (IP) with V5 showed three V5 related bands in testes but absent expression in females and in males lacking testes, which is identical to the expression pattern found with antibodies directed at dKPC2 **(Fig 3D and S3B Fig)**. The V5 tagged dKPC2 construct rescued fertility defects in dKPC2 mutants and thus is fully functional **(Fig 1G** and **S1 Data)**.

There are several post-translational protein modifications that could account for the dKPC2 isoforms detected on Western blots. The ~58 kDa doublet detected on Western was PNGase and EndoH resistant, indicating that dKPC2 is not glycosylated **(S4 Fig)**. dKPC2 is similar in domain architecture to the Yeast DNA repair protein RAD23 and its human homologues [32]. These proteins, which also contain UBL and UBA domains have been reported to be ubiquitinated and phosphorylated [33,34]. To test whether dKPC2 was ubiquitinated we treated testis lysates with deubiquitinase and found that both bands in the ~58kDa doublet shifted down by ~8.5 kDa, which approximates the size of a single ubiquitin molecule **(Fig 3E).** When we treated cell lysates with phosphatase, we converted the doublet to a single band **(Fig 3F)**. Taken together these data indicate that the larger band in the dKPC2 doublet is likely to be monoubiquitinated and phosphorylated while the smaller band is only monoubiquitinated. The 49kDa protein represents the un-modified isoform.

We sought to further characterize the expression pattern of dKPC2 by probing Western blots prepared from different portions of the male reproductive organs with anti-dKPC2 **(Fig 3G)**. All three dKPC2 related bands could be visualized in whole testes. In mature sperm dissected from the seminal vesicles, however, only the largest band in the doublet was detected, suggesting that the mature form of dKPC2 is the monoubiquitinated and phosphorylated species **(Fig 3G and S4 Fig)**. dKPC2 was not detected in the seminal vesicle capsule indicating that dKPC2 is a sperm specific protein. Staining of mature sperm from seminal vesicles with anti-dKPC2 revealed that the protein is located at the tip of the sperm tail and partially co-localizes with Amo **(Fig 3H and 3I)**. *dKpc2*^*1*^ mutant sperm did not stain with anti-dKPC2 supporting the specificity of the antibody staining pattern (Fig 3H). Despite their proximity in sperm dKPC2 and AMO did not show physical interaction in co-immunoprecipitation experiments **(S5 Fig)**.

### *Drosophila* KPC1 (dKPC1), an E3 Ligase, is also required for Amo localization

**Fig 4:**
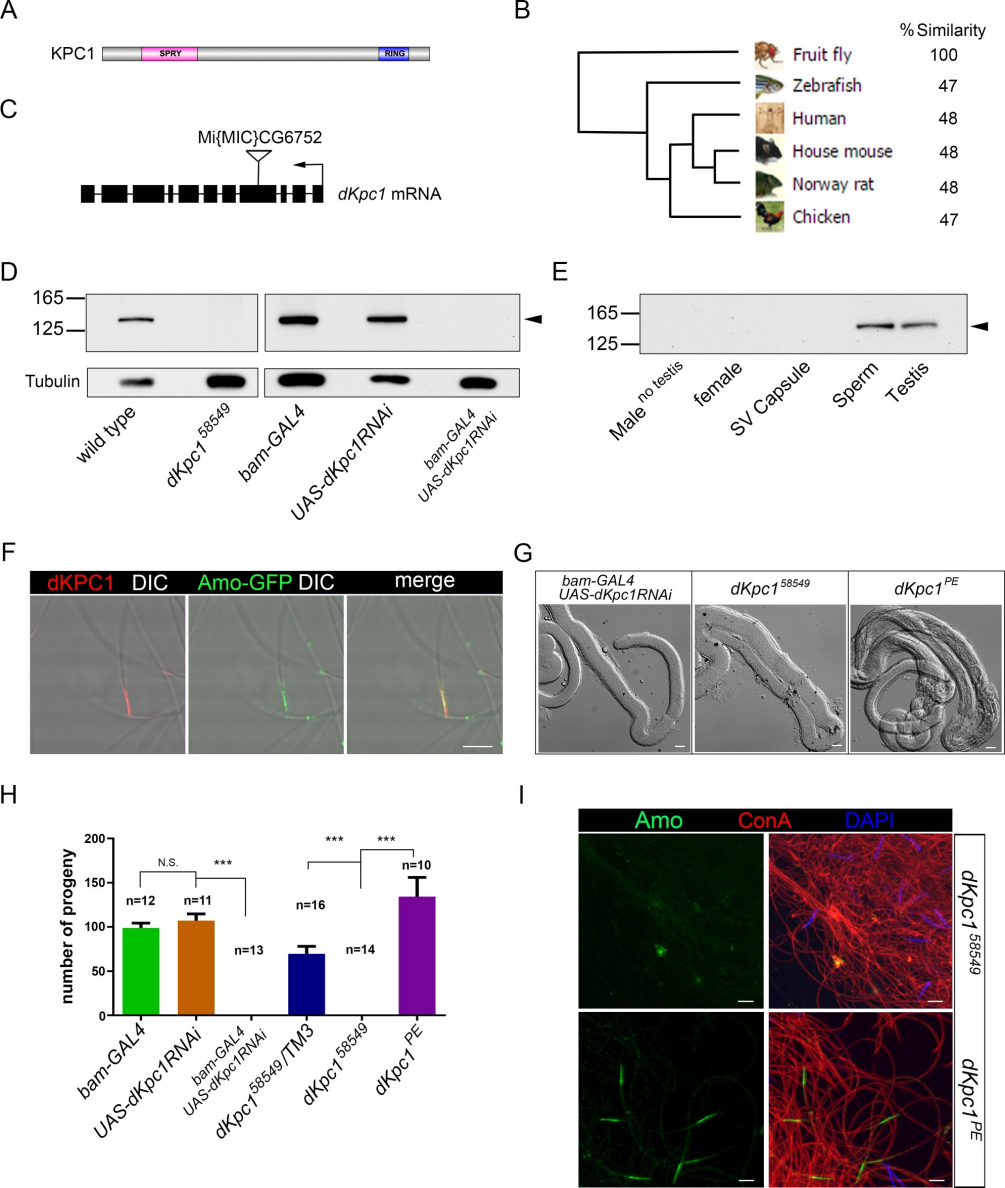
*Drosophila* Homolog of KPC1 (dKPC1) is required for sperm storage and Amo Localization. (A) Domain structure of dKPC1 showing an N-terminal SPRY domain and a C-terminal Ring-finger domain. dKPC1 has 1332 amino acids and is predicted to be ~151 kDa in size. (B) Phylogenetic tree demonstrates homology between dKPC1 and related proteins. (C) Map of the CG6752/*dKpc1* locus showing the MiMIC transposon insertion (*dKpc1*^*58549*^*)* in Exon 4. Black arrow indicates the direction of transcription. (D-E) Western blots probed with antisera to dKPC1. The antibody detects a single band of ~151 kDa, indicated by the arrowhead, in wild type testes and sperm. There is no dKPC1 detectable in testes dissected from *dKpc1*^*58549*^ males, from males with RNAi mediated knock-down of *dKpc1*, in females or in males lacking testes. (F) Amo-GFP sperm stained with antisera to dKPC1: red and to GFP: green. DIC and fluorescence images are merged showing that the two proteins partially co-localize at the tip of the sperm tail. Scale bars: 5 μm. (G) DIC images of wild type seminal receptacles dissected after mating with males of the indicated genotypes. Receptacles are empty after mating with *dKpc1*^*58549*^ males and males with RNAi-mediated knock-down of *dKpc1*. Sperm storage is rescued after precise excision of the transposon (*dKpc1*^*PE*^). Scale bars: 20 μm. (H) Fertility tests with males of the indicated genotypes mated to wild type females. *dKpc1*^*58549*^ males and males with RNAi-mediated knock down of *dKpc1* are sterile. Fertility is rescued by precise excision of the MiMIC transposon in *dKpc1* (*dKpc1*^*PE*^). The number of tests per genotype is denoted above the bars. ***p< 0.001. N.S. = not significant. (I) Wild type and *dKpc1*^*58549*^ sperm stained with anti Amo: green, concanavalin A: red and DAPI: blue. Amo is missing in *dKpc1*^*58549*^ sperm tails but localization is rescued by precise excision of the MiMIC transposon (*dKpc1*^*PE*^). Scale bars: 5 μm.

Vertebrate KPC2 forms part of the KIP1 Ubiquitination Promoting Complex (KPC), which includes the E3 ubiquitin ligase, KPC1[27,28,35]. To determine if there was a *Drosophila melanogaster* homolog of KPC1, we queried the *Drosophila* genome using human KPC1 and identified a homolog CG6752 (*dKpc1*) that was 30% identical and 48% similar to its human counterpart **(Fig 4A and 4B)**. *dKpc1* is predicted to encode a polypeptide with 1332 amino acids (~151 kDa), containing a RING-finger domain near its carboxy terminus, consistent with its role as an E3-ligase **(Fig 4A)**. The protein is also predicted to contain a SPRY motif, a domain of unknown function which is also found in the mammalian Ryanodine receptor [36].

We used two strategies to generate flies deficient in *dKpc1*. We identified a transgenic insertion in exon 4 of *dKpc1* (*dkpc1*^*58549*^, http://flystocks.bio.indiana.edu/Reports/58549.html) that is predicted to disrupt the gene **(Fig 4C)**. In addition, we used *bam-Gal4-VP16* to direct RNA interference (RNAi) knockdown of *dKpc1* in the male germ line (*bam-GAL4; UAS-dKpc1RNAi*). We raised a polyclonal rabbit antibody to dKPC1 that failed to detect the protein on testes Western blots from both *dkpc1*^*58549*^ and *bam-GAL4; UAS-dKpc1RNAi* males (**Fig 4D, S6A and S6B Fig)**. In addition, dKPC1 had an identical expression pattern to dKPC2 in wild type flies and is a male specific protein found primarily in sperm and absent in females **(Fig 4E and S3C Fig)**. Immunostaining of mature sperm with anti-dKPC1 showed that the protein co-localizes with Amo at the tip of the sperm tail but again we did not detect physical interaction *in vivo* **(Fig 4F, S5 and S6 Figs).**

dKPC1 mutant males were sterile due to a sperm storage defect **(Fig 4G and 4H** and **S1 Data)**. In addition, Amo staining was absent at the tip of the sperm flagellum (**Fig 4I and S6D Fig)**. In the case of *dkpc1*^*58549*^ we generated a line with precise excision of the P-element (*dKpc1*^*PE*^), which restored fertility, Amo localization in sperm and expression of dKPC1 **(Fig 4G–4I** and **S6A and S6E Fig** and **S1 Data)**.

### *Drosophila* KPC1/KPC2 form a complex *in vivo*

**Fig 5:**
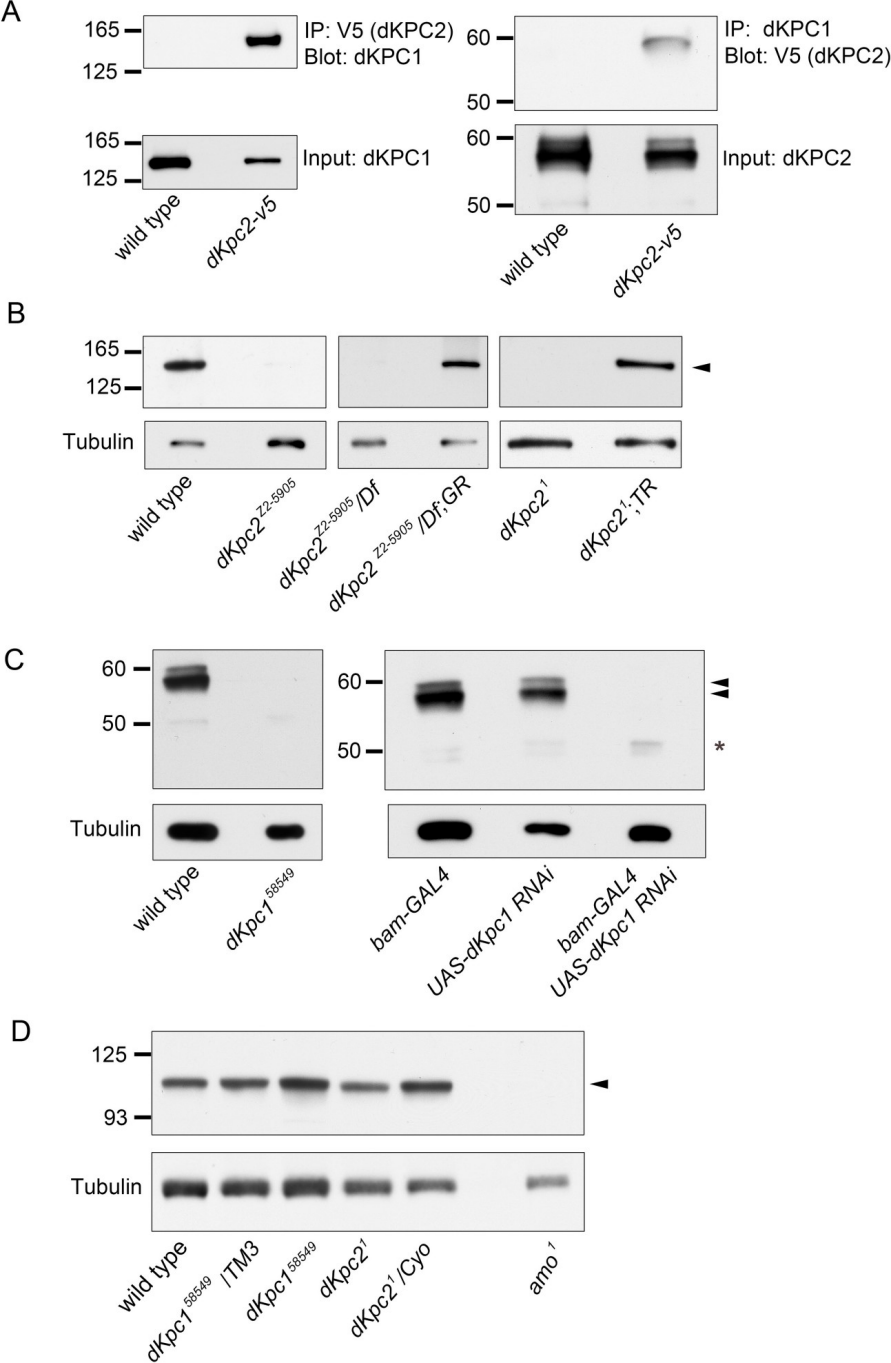
dKPC1 and dKPC2 form a complex in vivo. (A) Co-immunoprecipitation of dKPC1 and dKPC2. Extracts from testes expressing V5-tagged dKPC2 (dKPC2-V5) were immunoprecipitated with anti-V5 antibodies (left panel) or anti-dKPC1 (right panel). Immune complexes were fractionated and Western blots probed with anti-dKPC1 (left) or anti-V5 (right). Lower panels show corresponding immunoblots. Flies without a V5 tag served as a negative control. (B-C) Western blots prepared from testes lysates probed with anti-dKPC1 (B) or anti-dKPC2 (C). dKPC1 is indicated by an arrowhead in (B). The three dKPC2 isoforms in (C, see text) are indicated by arrowheads and an asterisk. dKPC1 is undetectable in dKPC2 mutant testes and the reverse is true in dKPC1 mutant testes. In each case absence of the protein can be rescued by re-expression of the missing component of the complex, either dKPC2 (B) or dKPC1 (C). (D) Western blots prepared from testes lysates of the indicated genotypes probed with anti-Amo. The band corresponding to Amo is indicated by an arrowhead. Loading control: Tubulin.

To test whether dKPC1 and dKPC2 form a complex, we conducted co-Immunoprecipitation (IP) experiments *in vivo* **(Fig 5A)**. In male flies bearing a V5-tagged dKPC2 transgene, IP with V5 could co-IP dKPC1. Conversely, anti-dKPC1 immunoprecipitated V5-tagged dKPC2. Interestingly, only the largest band in the dKPC2 58kDa doublet was detected in the dKPC1/KPC2 complex (**Fig 5A**).

We also found that dKPC1 was required for stabilization of dKPC2 and vice-versa **(Fig 5B and 5C)**. dKPC1 was absent in testes lysates from *dKpc2*^*1*^ mutant flies but present after transgenic re-expression of dKPC2 **(Fig 5B)**. Conversely, dKPC2 was barely detectable in *dKpc1*^*58549*^ mutants and in flies with testis-specific siRNA knock down of *dKpc1* **(Fig 5C and S7 Fig)**. Only the unmodified 49 kDa dKPC2 isoform, which was not co-IPed with dKPC1, could be visualized in *dKpc1*^*58549*^ mutant testes but the levels were grossly reduced in comparison with total dKPC2 levels in testes where the P-element had been precisely excised, suggesting that unmodified forms of dKPC2 undergo degradation (**S7 Fig** and **S1 Data**).

In the process of excising the Minos-mediated integration cassette (MiMIC) in *dKpc1*^*58549*^ we generated another *dKPC1* allele (*dKpc1*^*1*^) with a 6-base pair insertion in the protein’s SPRY domain **(S8A–S8D Fig)**. This protein is synthesized and can be detected in testes lysates **(S8B Fig)** but does not interact with dKPC2 **(S8C Fig)** and does not localize to the tip of the sperm tail **(S8D Fig)**. Amo sperm localization is also missing in this mutant **(S8D Fig)**. Interestingly there is no detectable dKPC2 ubiquitination in *dKpc1*^*1*^ mutant testes and the level of the unmodified, 49 kDa band appears to be increased. We conclude that dKPC1 is the likely E3-ligase for dKPC2 and that the interaction between the two proteins is also required for the stability of the complex.

One possible explanation for defective Amo localization in *dKpc1* and *dKpc2* mutant sperm is increased degradation. We assayed total Amo expression by Western blot analysis of whole testis lysates. We found that total Amo expression levels were preserved in both *dKpc1* and *dKpc2* mutant testes **(Fig 5D).** We were unable to show convincingly that Amo itself is ubiquitinated.

## Discussion

The polycystin complex is expressed in cilia and disruption of its ciliary localization results in polycystic kidney disease in humans and diverse phenotypes in other organisms [8,10,37,38]. Similarly, in *Drosophila melanogaster* the PC2 homologue, AMO, must reach the tip of the sperm flagellum for proper sperm storage in female flies [12]. In the current study, we conducted a forward genetic screen in a collection of male sterile mutant flies, looking for mislocalization of Amo. We identified an EMS mutant harboring a truncating mutation in the Drosophila homologue of mammalian KPC2 (*dKpc2*), which lacked Amo at the tip of the sperm flagellum. We confirmed the phenotype in a second *dKpc2* knock out allele that we generated by homologous recombination. Mammalian KPC2 is a component of the KIP1 ubiquitination-promoting complex (KPC), which is a heterodimer composed of KPC2 (aka *UBAC1*) and an E3 ligase, KPC1 (*Rnf123*, 27–29]. Using a candidate approach we found that flies lacking a predicted Drosophila homologue of human KPC1 (*dKpc1*) likewise were missing appropriate Amo localization at the tip of the sperm tail. In addition, *dKpc1* and *dKpc2* mutant males phenocopied the loss of *Amo* and were sterile due to a sperm storage defect.

There is relatively little known about the function of mammalian KPC *in vivo* and to date no knock-out mouse models of either component of the complex has been reported. KPC1 is a ring finger containing protein that functions as an E3 ligase whereas its binding partner, KPC2 is an adaptor, containing an ubiquitin like domain (UBL) at its N-terminus and two C-terminal ubiquitin associated domains (UBA)[27]. UBL motifs have structural homology to ubiquitin while the UBA domains bind ubiquitin [39]. KPC2 not only stabilizes KPC1 at the protein level, but in certain instances may inhibit its ubiquitinating activity [27,35]. In cell culture studies, KPC2 binds to polyubiquitin and the 26S proteasome, presumably delivering ubiquitinated proteins to the proteasome for degradation [28]. One target of the KPC complex is the cyclin-dependent kinase p27Kip1, which is a negative regulator of cell cycle progression [27, 40]. KPC1 ubiquitinates p27Kip1 and thereby promotes its cytoplasmic degradation at the G0-G1 transition [27–29]. A recent study has also revealed a novel role for KPC1 as the E3 ligase responsible for the limited proteasomal processing of the NF-κB1 precursor p105 to its active p50 subunits [35,41]. The p50 homodimers have a distinct anti-proliferative transcriptional profile. Over expression of p50 and KPC1 are associated with diminished growth of xenograft tumors whereas they are down regulated in several malignancies including melanoma, glioblastoma and squamous cell cancers of the head and neck [35,41,42].

The unbiased screen reported herein uncovered an unexpected role for the KPC complex in male fertility in flies, presumably by targeting Amo to a specialized area of the sperm flagellar membrane. Consistent with this finding, both dKPC1 and dKPC2 are detected by Western blot only in male flies and the complex is highly sperm enriched. Sperm staining with cognate antibodies confirmed that both dKPC1 and dKPC2 are localized in a very restricted zone at the tip of the flagellum. Similar to the vertebrate KPC complex, co-IP experiments showed that dKPC1 and dKPC2 physically interact and stabilize one another in a reciprocal fashion. dKPC1 was largely absent in dKPC2 knock out testes and the reverse was also true. To our knowledge, the vertebrate KPC complex has not been previously associated with either cilia or flagella but other proteins in the ubiquitin conjugating system have been identified in both the *Chlamydomonas* flagellar proteome and the proteome of the mouse photoreceptor sensory cilium and sperm [43–46]. Moreover, ubiquitination has been implicated in flagellar disassembly and signal transduction in *Chlamydomonas*, ciliary assembly and axoneme extension in RPE cells and spermatogenesis in mice [45,47–49].

Our biochemical studies also revealed that dKPC2 undergoes a series of post-translational modifications *in vivo* that have not been described for its mammalian counterparts. Using rigorously validated antibodies, we found that dKPC2 existed in 3 distinct isoforms in the Drosophila testis: unmodified, monoubiquitinated and phosphorylated-monubiquitinated. Mature sperm in the seminal vesicle contain only the phosphorylated, monoubiquitinated protein and this is the form that appears to co-IP with dKPC1. This is consistent with the idea that the fully modified protein represents the mature and perhaps the fully functional form of dKPC2. Our data also strongly suggest that dKPC1 is the E3 ligase that monoubiquitinates dKPC2. A *dKpc1* allele with a two-amino acid, in frame Spry domain deletion results in an expressed form of dKPC1 that fails to interact with dKPC2. dKPC2 completely lacks monoubiquitination in the testes of this mutant and Amo is also mislocalized.

It seems likely that these post-translational dKPC2 modifications regulate protein-protein interactions since this has been shown for other members of the UBA/UBL family of ubiquitin binding proteins. For example, NMR studies of the yeast RAD23 human homologues, hHR23a and hH23b, suggest that weak intramolecular interactions occur between their UBL and UBA motifs [50,51]. One model proposes that these weak interactions prevent binding to the proteasome or other ubiquitinated substrates [39]. Under favorable conditions, however, UBL-UBA interactions are disrupted, permitting additional protein-protein interactions. In the case of dKPC2, monoubiquitination or phosphorylation could disrupt intermolecular binding between its UBA and UBL motifs, allowing UBL to bind with other protein interactors. Alternatively, monoubiquitination could result in internal coupling to the UBA domain thereby assuming an auto inhibited confirmation. Additional study will be required to further characterize and define the role of specific dKPC2 protein modifications including the identity of the kinase that phosphorylates dKPC2 and the impact of individual modifications on Amo localization.

Although we cannot entirely exclude a transient interaction between the *Drosophila* KPC ubiquitinating complex and Amo, the mechanism by which dKPC1 and dKPC2 participate in proper trafficking of Amo to the sperm tail appears to be indirect. Although dKPC1 and dKPC2 partially co-localize with Amo in sperm, we were unable to demonstrate direct binding between Amo and either dKPC1 or dKPC2 in co-immunoprecipitation studies. We tested whether the dKPC complex might be involved in post-translational cleavage of Amo as was described for NF-κB1[35,41]. In *Chlamydomonas reinhardtii* Pkd2 is cleaved into two fragments and only the cleaved products are found in flagella [52]. However, we were unable to confirm that either native Amo or over expressed tagged versions of Amo are cleaved. We also tested whether Amo might be degraded in *dKpc1/2* mutant testes. This seemed like a reasonable hypothesis since KPC2 has been shown to stabilize proteins by dampening the E3-ligase activity of KPC1 [27,53]. However, the levels of Amo in various *dKpc1* and *dKpc2* mutant testes were similar to wild type, excluding the possibility that the Drosophila KPC complex is required to prevent Amo degradation. In addition, we were unable to convincingly show that Amo is ubiquitinated *in vivo*. Taken together our findings point to a model whereby the KPC complex is likely to interact with unidentified proteins that are involved in the final stages of targeting Amo to a membrane domain at the tip of the sperm flagellum. This could conceivably occur through modifications of the sperm tail membrane that are necessary for Amo localization. Interestingly monoubiquitinated adaptor proteins similar in structure to KPC2 are known to participate in membrane protein transport (reviewed in [54]). We have developed a series of validated reagents, including antibodies and transgenic fly lines, that can now be used to immunopurify KPC complex interactors, which may form part of the Amo trafficking complex.

In summary, we have used a genetic screen in *Drosophila* to identify a role for the KPC1/KPC2 ubiquitination complex in male fertility. In *Drosophila*, these proteins are associated with the sperm tail and are required for the localization of Amo to the tip of the sperm flagellum. It will be interesting to determine whether the KPC1/2 complex plays a conserved role in localizing proteins to vertebrate cilia and or flagella.

## Materials and methods

### Fly stocks

The following stocks were obtained from the Bloomington *Drosophila* Stock Center: MiMIC insertion in CG672 (stock # 58549), MiMIC transposase (stock # 24613), GFP tagged protein disulfide isomerase (stock # 6839), CG6752 RNAi line (stock # 64019). Wild type flies were *w*^*1118*^. The collection of male sterile EMS mutants was previously described and was derived from the Zuker collection [30,31]. The subset of 60 male sterile fly stocks producing mature sperm along with a stock for Chromosome 2 meiotic mapping were provided by Dr. Barbara Wakimoto. The *bam-Gal4-VP16* line was provided by Helen White-Cooper [55]. The *Amo* mutant flies and a line expressing Protamine-B-DS-Red and don-juan-GFP on chromosome 3 were previously described [11,12]. All flies were reared according to standard procedures and maintained at 25°C.

### Phylogenetic trees

The evolutionary molecular analyses in Fig 1 and Fig 4 were generated by TreeFam (Tree families database) at http://www.treefam.org/search/sequence [56]. The trees were simplified to show the common animal models. The homology values were retrieved from Homogene at NCBI (https://www.ncbi.nlm.nih.gov/homologene).

### RT-PCR

We extracted total RNA from flies with TRIzol reagent according to the manufacturer’s protocol (Invitrogen). Total RNA (2 μg) was treated with RNase-free DNase and then used for first strand cDNA synthesis with Superscript II reverse transcriptase (Invitrogen). A reverse transcriptase negative control was included for each sample. The location of primers for transcripts A, B and C are shown in S2A Fig. The following primers were used:

#### Transcript A

105-AF: 5’-AGTTGCGTCCTAAGGACTCCAT-3’ and 105-AR: 5’-GCGATTTGTATGGGCTGATTTC-3’ under the following PCR conditions: 94°C for 5 minutes, 35 cycles of 94°C for 30 s, 53°C for 30 s, 72°C for 30 s and a final elongation step of 72°C for 10 min. When these primers are used, the genomic DNA product is ~378 base pairs (bp) and the cDNA product is ~180 bp.

#### Transcript B

105-BF: 5’-TTTGTCTTTCGAAATTCTCCGTTGG-3’ and 105-BR: 5’-GGATTAAGATGCCGCACATC-3’ under the following PCR conditions: 94°C for 5 minutes, 35 cycles of 94°C for 30 s, 53°C for 30 s, 72°C for 30 s and a final elongation step of 72°C for 10 min. The genomic DNA product is ~398 bp and the cDNA product is ~334 bp.

#### Transcript C

105-CF: 5’-AGTTGCGTCCTAAGGACTCCAT-3’ and 105-CR: GGCTGATTTCCTGAGAAACAAA-3’ under the following PCR conditions: 94°C for 5 minutes, 35 cycles of 94°C for 30 s, 53°C for 30 s, 72°C for 30 s and a final elongation step of 72°C for 10 min. When these primers are used, the genomic DNA product is ~289 bp and the cDNA product is ~167 bp.

#### rp49 (Rpl32, CG7939)

was used as a loading control and amplified using the following primers: Forward: 5’-GCGCACCAAGCACTTCATC-3’ and Reverse” 5’-GACGCACTCTGTTGTCGATACC-3’ under the following PCR conditions: 94°C for 5 minutes, 35 cycles of 94°C for 30 s, 55°C for 30 s, 72°C for 30 s and a final elongation step of 72°C for 10 min. These primers amplify a band of 100 bp.

### Generation of *dkpc2*^*1*^ mutant

Briefly we used ends out homologous recombination to generate a *dKpc2* mutant allele that contained a **~**1kb nucleotide deletion, spanning the translation initiation codons of all *dKpc2* isoforms [57]. To make the targeting vector, we amplified ~3 Kb genomic fragments at the 5’ and 3’ ends of the *dKpc2* region from a BAC clone CH322-39H04 (BACPAC Resource Center). The primer sequences for the 5’ homologous arm were: Forward, 5’-TATCCCTAGGGGATCCACAAAGGCCTCTTCCACCGA-3’ and Reverse, 5’TGGTACCTTTGGATCCTTGGGTCTGCAGCTAGGTGTTTGT-3’ (Fragment size 2990 bp). The primer sequences for the 3’ homologous arm were: Forward, 5’-GCATGCAAAGCGGCCGCCTAATCCTATTCCGTTTCCAGATCCAA-3’ and Reverse, 5’-CCGCGGAAAGCGGCCGCACACGACTCTTCAATAACACACACC-3’ (Fragment size 3056 bp). The 5’ and 3’ arms were cloned into the pw35GAL vector using BamH1 and Not1, respectively. Transgenic flies were generated by germ line transformation (BestGene Inc., Chino Hills CA). We obtained homologous recombinants as previously described [57]. We confirmed gene targeting by PCR from genomic DNA using primer pair 1 (Fig 2): Forward, 5’- TCCTCGTTGAATGTCCACAC-3’ and Reverse, 5’-GACGGTATCGCGGATTAAGA-3’. We confirmed the absence of cDNA transcripts using primer pair 2 (Fig 2): Forward, 5’-CTTCCATCTCACCCTCCACGATTG-3’ and Reverse, 5’-GACTTTTTTGGCATTTCTGGG-3’.

### Generation of *dKpc2* transgenic flies

To obtain a *dKpc2* (CG11025) rescue construct, a 6.3 Kb genomic fragment containing the native (*dKpc2*) promoter elements and 250 bp of 5’ sequence from the neighboring gene (CG9854) was amplified using the following primers: Forward, 5’-TAGCATCTAGATTGACCCACTGCTTGACC-3’ and Reverse, 5’-TAGCAGTTAACCAACATGCAACCGCA-3’. We also added a V5 tag to this construct by amplifying sequences encoding the 14-amino acid V5 linker (GKPIPNPLLGLDST) from pcDNA 3.1/V5-His TOPO. This was cloned in frame at the end of the C-terminal *dKpc2* exon (before the stop codon) using a three step PCR method [58]. The C-terminal region was sequenced to confirm that the V5 linker was cloned in frame. Both tagged and un-tagged fragments were cloned into the pATTB vector and transgenic flies were generated using PhiC31 integrase-mediated transgenesis on the third chromosome to minimize position effect (Bloomington stock number 24749) [59].

For testis-specific expression of the *dKpc2*, Transcript B was amplified from a cDNA clone (clone # AT01875, *Drosophila* Genome Resource Center, NIH 2P40OD010949) using a high-fidelity PCR kit (Phusion HF, New England Biolabs, Cat# M0530S) and the following primers: Forward, 5’-GAATTCATGCAAAAACTGCGCAGTTTGTTTC-3’ and Reverse, 5’-GTTAACTCACCAGCGGTTCGTATAGAAC-3’. The resulting 1308 bp cDNA product was sequenced and then cloned into a modified pGMR vector, where the β2-tubulin promoter replaced the glass promoter elements (gift from John Belote, Syracuse University and Kate Beckingham, Rice University). Transgenic flies were generated by standard P element-mediated germ-line transformation (BestGene Inc., Chino Hills CA).

### Generation of GFP tagged *Amo* transgenic flies

Since our antibodies were all rabbit polyclonals, we generated GFP tagged AMO transgenic flies, which allowed us to use mouse monoclonal anti-GFP for co-localization studies. The Amo genomic rescue construct has been previously described [11,12]. To introduce a green fluorescent protein (GFP) tag in frame, GFP was amplified from pcDNA3-EGFP. This fragment was cloned in frame before the Amo stop codon using three-step PCR [58]. The construct in the pATTB vector was used to generate transformants using PhiC31 integrase-mediated transgenesis on the third chromosome (Bloomington stock number 24749). This line fully rescued the *amo* null phenotype.

### P-element mobilization

We generated a precise excision of the Minos insertion *MiMIC dKPC1*^*MI12673*^ (Bloomington Stock 58549) according to Metaxakis et al [60]. DNA was prepared from flies that had lost the y+ marker and screened using the following primers: DNA was prepared from flies that had lost the *y+* marker and screened using the following primers: Forward, 5’-GGCAAATATGCAGTACACGGT-3’ and Reverse, 5’-TATAATTGCTTTCCTAGGTACGGC-3’. The PCR product was characterized further by DNA sequencing.

### Antibody generation

cDNA clones for *dKpc1* and *dKpc2* were ordered from the *Drosophila* Genome Resource Center (DGRC, NIH grant 2P40OD010949, clone # AT11570; Stock Number 18250 and clone # AT01875; Stock Number 1017499). For *dKpc2*, the entire coding region of transcript B was cloned into the pHAT2 vector (European Molecular Biology Laboratory, gift from Paul F. Worley, Johns Hopkins University). For *dKpc1*, a fragment encoding amino acids 1063 to 1306 including the Ring Domain was amplified from the cDNA clone and then sub-cloned into pHAT2. The His-tagged fusion proteins were expressed in *E*. *coli* (*BL21*, Agilent), purified with nickel resin (Ni-NTA, QIAGEN, Catalog # 30210) and injected into rabbits (Covance, Inc).

### Western blotting and co-immunoprecipitation

Testes from 3-5-day old male flies were homogenized in lysis buffer (0.5% Triton X-100, 20mM Tris pH = 7.5, 50mM NaCl, 50mM, NaF, 15mM Na_4_P_2_O_7_, 1mM EDTA pH = 8 and complete protease inhibtor cocktail (Roche). The lysates were used to prepare Western blots that were probed with the following antibodies: anti-dKPC2 1:1000 dilution, anti-dKPC1 1:1000 dilution, anti-tubulin 1:1000 dilution (E7, Hybridoma Bank, University of Iowa), anti-V5 (ThermoFisher, Catalog # R960-25), anti-Amo 1:1000 dilution [11]. To test for ubiquitination lysates were treated with de-ubiquitinase (USP2core, LifeSensors, Catalog # DB501) at 37°C degree for 30 minutes. To test for phosphorylation lysates were treated with Lambda Protein Phosphatase (New England BioLabs, Catalogue # P0753S).

To perform co-immunoprecipitation experiments, 50 pairs of testes, dissected from 3-5- day old flies, were homogenized in lysis buffer on ice for 30–60 minutes and centrifuged at 10, 000 RPM. The cleared lysates were incubated with protein G Sepharose beads for 1 hour at 4°C to reduce nonspecific binding. Then lysates (1ml) were incubated with 2 μl of anti-dKPC1, 2 μl anti-dKPC2, 2 μl of anti-V5 (ThermoFisher, Catalog # R960-25) or 1 μl anti-GFP (ThermoFisher, Catalog # A-11122) for approximately 12 hours at 4°C and then with Protein G Sepharose beads. The immunoprecipitation products were fractionated by SDS-PAGE and blotted with appropriate antibodies as above.

### *Drosophila* immunofluorescence/histology

Dissection and preparation of testis and sperm have been previously described except that testis and sperm were fixed for 10 minutes at room temperature in Cytofix Fixation Buffer (BD biosciences, Catalog # 554655) [11, 12]. The following primary antibodies were used: rabbit anti-Amo (1:1000 for sperm and 1:2000 for testes), rabbit anti-dKPC1 (1:500), rabbit anti-dKPC2 (1: 500), mouse monoclonal anti-GFP (1:50, Sigma). Secondary fluorescent antibodies were Alexa fluor 488 conjugate goat anti-rabbit and Alexa fluor 555 conjugate goat anti-rabbit (1:1000; Molecular Probes). Sperm tails and heads were visualized with Alexa fluor 594 conjugated concavalin A (1:20, 50 μg/ml Molecular Probes) and 4′,6-Diamidin-2-phenylindol (DAPI), respectively. Images were recorded using a Zeiss LSM510 confocal microscope (Zeiss, Germany).

### Male fertility assays

Males of various genotypes were separated upon eclosion and maintained in isolation 3 days prior to mating. Single males were mated with two *w*^*1118*^ virgin females for 5 days. At that time both parents were removed from the vial. The number of progeny that eclosed from each vial was counted. A minimum of 10 vials were scored for each genotype.

### Statistics

Data are presented as mean values ± s.e.m. (N = number of experiments, n = observations within an experiment). Unpaired student's t-Test was used for statistical analysis between two groups.

## Supporting information

S1 Fig Characterization of Z2-5905 mutant phenotype. (A-D) Amo localization in wild type (A, C) and Z2-5905 testes (B, D). Anti-Amo: red, Phalloidin: green, DAPI: blue. Scale bars: 10 μm. White arrows indicate investment cones (IC). Amo localization is not altered in Z2-5905 mutant testes. (E-F) DIC images of wild type seminal receptacles dissected after mating with wild type (E) or Z2-5905 mutant males (F). Seminal receptacles are empty after mating with Z2-5905 males. Scale bars: 20 μm. (G-H) Testes dissected from wild type (G) and Z2-5905 males (H). Sperm heads and tails labeled by *Prot-B-DsRed* (red) and *dj-GFP* (green) transgenes, respectively. DIC and fluorescence images are superimposed. There are similar numbers of mature sperm. Scale bars: 100 μm. (I-N) Testes from PDI-GFP flies were stained with Anti-Amo (red). Amo co-localizes with the ER marker PDI in primary spermatocytes in both wild type and Z2-5905 flies. Scale bars: 10 μm.
(TIF)

S2 Fig Z2-5905 mutant has a splice site mutation in the *Drosophila* homolog of KPC2 (*dKpc2*) that disrupts testis expression. (A) Physical map of the Z2-5905 locus. Deficiency mapping narrowed the mutation in Z2-5905 to a small region on Chromosome 2R contained in two overlapping deficiencies, ED3728 and BSC594. The genes mapping to the interval are denoted by black arrowheads. Genomic sequencing identified a G to A transition in CG11025 that affects a canonical splice donor site as indicated. CG11025 is predicted to encode three transcripts (A, B and C) with coding exons indicated in green and non-coding exons in black. The mutation in the canonical splice donor site affects splicing of a small exon contained only in transcripts B and C. Black arrows show PCR primers used for RT-PCR in (C-E). (B) Sanger sequencing from genomic DNA of Z2-5905 showing the G/C to A/T transition indicated by the arrow. (C) RTPCR for transcript B using the indicated primers. The genomic band (gDNA) is ~398 base pairs (bp) while the cDNA band is ~ 334 bp. Transcript B is present in males and in testis but not females or the male body with testes dissected away. In the Z2-5905 mutant, splicing is disrupted as shown by the ~398 bp band, which was sequenced to confirm read-through. The RT-PCR product is absent in the RT negative control. (D) RT-PCR for Transcript C using the indicated primers. The genomic (gDNA) band is ~ 289 base pairs. The cDNA band of ~ 167 bp, is detected in males and females but absent in Z2-5905 mutant males. (E) RT-PCR for Transcript A using the indicated primers. These primers also amplify transcript C. The gDNA band is ~378bp. Sequencing the band visualized in WT testis reveals that it is Transcript C. There is no fragment amplified in Z2-5905 testis. The data is summarized in S1 Table.
(TIF)

S3 Fig dKPC2 and dKPC1 are male specific proteins. (A) Lysates were prepared from wild type and *dKpc2*^*1*^ male flies as indicated. Western blots were probed with anti-dKPC2. dKPC2 is easily detected in as little as one wild type male fly but absent in *dKpc2*^*1*^ males. (B) Extracts were prepared from male (M) or female (F) flies of the indicated genotypes and immunoprecipitated with mouse anti-V5 antibodies. Western blots were probed with anti-dKPC2 and goat anti-mouse IgG light chain as a loading control. dKPC2-V5 was only detected in male flies bearing the V5 tagged-*dKpc2* genomic construct but not in wild type males or females having *V5-dKpc2*. (C) Lysates were prepared from wild type male (M) and female (F) flies. Western Blots were probed with anti-dKPC1. Loading control: Tubulin. dKPC1 protein is not detected in female flies. In panels A and B, the three dKPC2 isoforms are indicated by arrowheads and an asterisk. In panel C the arrowhead indicates the dKPC1 band.
(TIF)

S4 Fig dKPC2 is not glycosylated. Total lysates from testis or sperm were treated with PNGaseF or EndoH as indicated. Western blots were probed with anti-dKPC2. dKPC2 resolves as 3 bands in testes but only the mature form is detected in sperm (see text) and there is no difference upon treatment of the lysates with either PNGaseF or EndoH. Black arrowheads indicate the dKPC2 doublet at ~58kDa. The asterisk denotes the unmodified product of Transcript B at ~49kDa.
(TIF)

S5 Fig dKPC1 and dKPC2 do not co-Immunoprecipitate (co-IP) with Amo. Extracts from Testes expressing Amo tagged at its C-terminus with GFP were immunoprecipitated with anti-GFP. Immune complexes were used to prepare Western blots that were probed with anti-GFP (left panel), anti-dKPC2 (middle panel) or anti-dKPC1 (right panel). Neither dKPC2 or dKPC1 co-IP with Amo. Amo-GFP is detected in the IP products. The Amo-GFP band, indicated by the arrowhead, is larger than the size of native protein (~105 kDa) due to the GFP tag.
(TIF)

S6 Fig Characterization of *dKpc1* mutant flies. (A-B) Western blots of testes lysates probed with anti-dPKC1 (arrowhead). There is no detectable dKPC1 in flies with the MiMIC transposon insertion in *dKpc1* (CG6752) exon 4 (*dKpc1*^*58549*^) or in flies harboring the transposon in *trans* with a deletion (Df) that completely removes *dKpc1*. Precise excision of the transposon results in re-expression of dKPC1 (*dKpc1*^*PE*^). Tubulin serves as a loading control. (C) Sperm stained with anti-dKPC1: green, concanavalin A: red, DAPI: blue. dKPC1 staining is negative in sperm with RNAi mediated knock-down of dKPC1. Scale bars: 5 μm. (D) Sperm stained with anti-Amo: green, concanavalin A: red, DAPI: blue. Amo is missing from the sperm tail in flies with RNAi mediated knock-down of dKPC1. RNAi expression in the testis was driven by Bam-Gal4. Scale bars: 10 μm. (E) Fertility tests with males mated to wild type females. *dKpc1*^*58549*^ males are sterile but fertility is rescued by precise excision of the transposon (*dKpc1*^*PE*^). The number of tests per genotype is denoted above the bars. *** P< 0.001.
(TIF)

S7 Fig dKPC1 is required for ubiquitination and stability of dKPC2. (A) Western blot prepared from testes lysates probed with Anti-dKPC2. The predominant dKPC2 isoform resolved in *dKpc1*^*58549*^ testes is the ~49 kDa, unmodified protein, indicated by the asterisk). Precise excision of the transposon (*dKpc1*^*PE*^) yields testes expression of all three isoforms (arrowheads and asterisk) but the amount of the unmodified isoform (asterisk) appears to be reduced. Tubulin serves as a loading control. (B) Quantification of the ~49kDa dKPC2 isoform. The ratio of the dKPC2 ~49 kDa isoform of dKPC2 to Tubulin was calculated in *dKpc1*^*58549*^ testes and normalized to *dKpc1*^*PE*^. The amount of unmodified dKPC2 in *dKpc1*^*58549*^ mutant testes was ~4 times that in control. Graph shows quantification of 3 independent experiments. *** P< .001.
(TIF)

S8 Fig The dKPC1 Spry domain is required for ubiquitination. (A) Schematic of the *dKpc1*^*1*^ allele, generated during transposase mediated excision, that contains a 6-base pair insertion in the predicted dKPC1 Spry domain. (B) Testes lysates were used to prepare Western blots that were probed with anti-dKPC1 (top panel) and anti-dKPC2 (middle panel). Tubulin serves as a loading control (lower panel). dKPC1 protein is still made in the *dKpc1*^*1*^ allele but there are no ubiquitinated dKPC2 isoforms. Only the ~49 kDa isoform is present (asterisk). (C) Extracts from testes were immunoprecipitated with anti-dKPC2 and western blots were probed with anti-dKPC1 (arrowhead). dKPC2 and dKPC1 do not co-IP in the *dKpc1*^*1*^ mutant, suggesting that ubiquitination is required for the interaction. (D) *dKpc1*^*1*^ mutant sperm stained with anti-dKPC1 (green, top row), anti-dKPC2 (green, middle row) and anti-Amo (green, bottom row) along with concanavalin A: red and DAPI: blue. *dKpc1*^*1*^ mutant sperm lack staining for all three proteins. Scale bars: 10 μm.
(TIF)

S1 Table Summary of RT-PCR data. Total RNA was extracted from male and female heads, bodies and relevant reproductive organs and used for cDNA synthesis. Primer pairs as indicated in S2A Fig were used for RT-PCR and the data was summarized in table form. n/a: not applicable. See Material and Methods for details.
(XLSX)

S1 Data Raw numerical data for all the figures. (XLSX)
