# Supplementary figures and images for: A genetic screen in Drosophila reveals an unexpected role for the KIP1 ubiquitination-promoting complex in male fertility
Source: PLoS Genet. 2020 Dec 30;16(12):e1009217. doi: 10.1371/journal.pgen.1009217 (PMC7802972; doi:10.1371/journal.pgen.1009217)

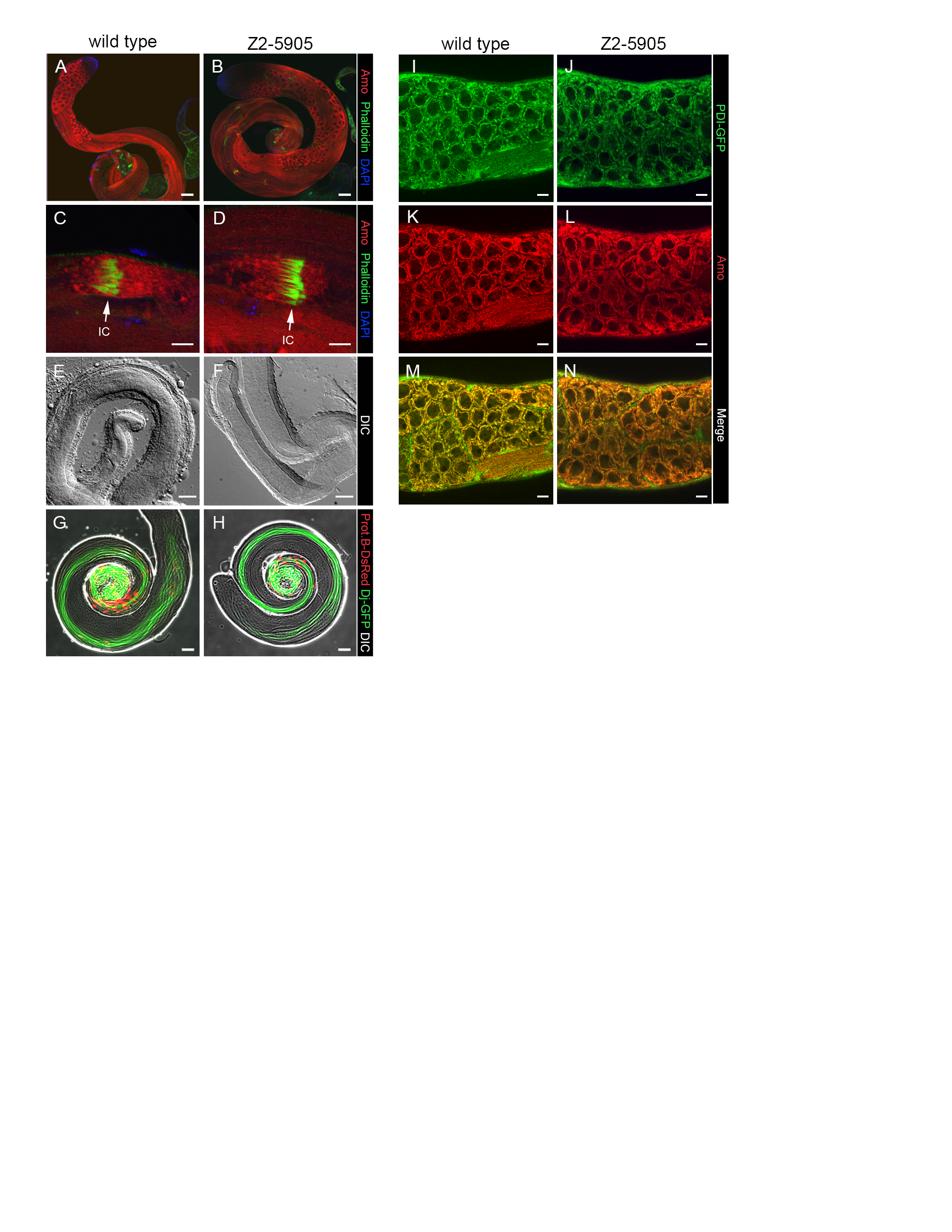

Supplement: S1 Fig — (A-D) Amo localization in wild type (A, C) and Z2-5905 testes (B, D). Anti-Amo: red, Phalloidin: green, DAPI: blue. Scale bars: 10 μm. White arrows indicate investment cones (IC). Amo localization is not altered in Z2-5905 mutant testes. (E-F) DIC images of wild type seminal receptacles dissected after mating with wild type (E) or Z2-5905 mutant males (F). Seminal receptacles are empty after mating with Z2-5905 males. Scale bars: 20 μm. (G-H) Testes dissected from wild type (G) and Z2-5905 males (H). Sperm heads and tails labeled by Prot-B-DsRed (red) and dj-GFP (green) transgenes, respectively. DIC and fluorescence images are superimposed. There are similar numbers of mature sperm. Scale bars: 100 μm. (I-N) Testes from PDI-GFP flies were stained with Anti-Amo (red). Amo co-localizes with the ER marker PDI in primary spermatocytes in both wild type and Z2-5905 flies. Scale bars: 10 μm. (TIF) [file pgen.1009217.s001.tif]

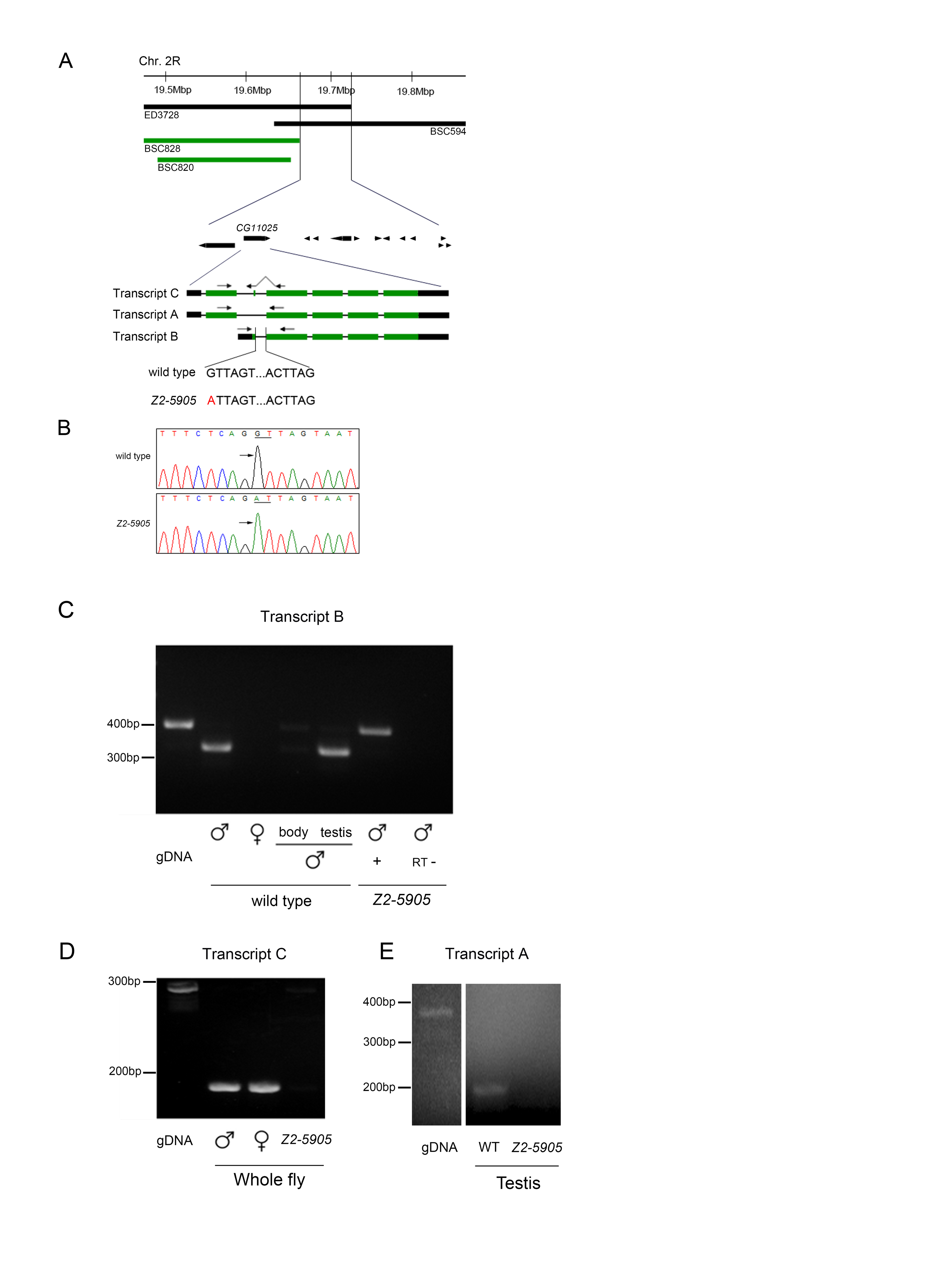

Supplement: S2 Fig — (A) Physical map of the Z2-5905 locus. Deficiency mapping narrowed the mutation in Z2-5905 to a small region on Chromosome 2R contained in two overlapping deficiencies, ED3728 and BSC594. The genes mapping to the interval are denoted by black arrowheads. Genomic sequencing identified a G to A transition in CG11025 that affects a canonical splice donor site as indicated. CG11025 is predicted to encode three transcripts (A, B and C) with coding exons indicated in green and non-coding exons in black. The mutation in the canonical splice donor site affects splicing of a small exon contained only in transcripts B and C. Black arrows show PCR primers used for RT-PCR in (C-E). (B) Sanger sequencing from genomic DNA of Z2-5905 showing the G/C to A/T transition indicated by the arrow. (C) RTPCR for transcript B using the indicated primers. The genomic band (gDNA) is ~398 base pairs (bp) while the cDNA band is ~ 334 bp. Transcript B is present in males and in testis but not females or the male body with testes dissected away. In the Z2-5905 mutant, splicing is disrupted as shown by the ~398 bp band, which was sequenced to confirm read-through. The RT-PCR product is absent in the RT negative control. (D) RT-PCR for Transcript C using the indicated primers. The genomic (gDNA) band is ~ 289 base pairs. The cDNA band of ~ 167 bp, is detected in males and females but absent in Z2-5905 mutant males. (E) RT-PCR for Transcript A using the indicated primers. These primers also amplify transcript C. The gDNA band is ~378bp. Sequencing the band visualized in WT testis reveals that it is Transcript C. There is no fragment amplified in Z2-5905 testis. The data is summarized in S1 Table. (TIF) [file pgen.1009217.s002.tif]

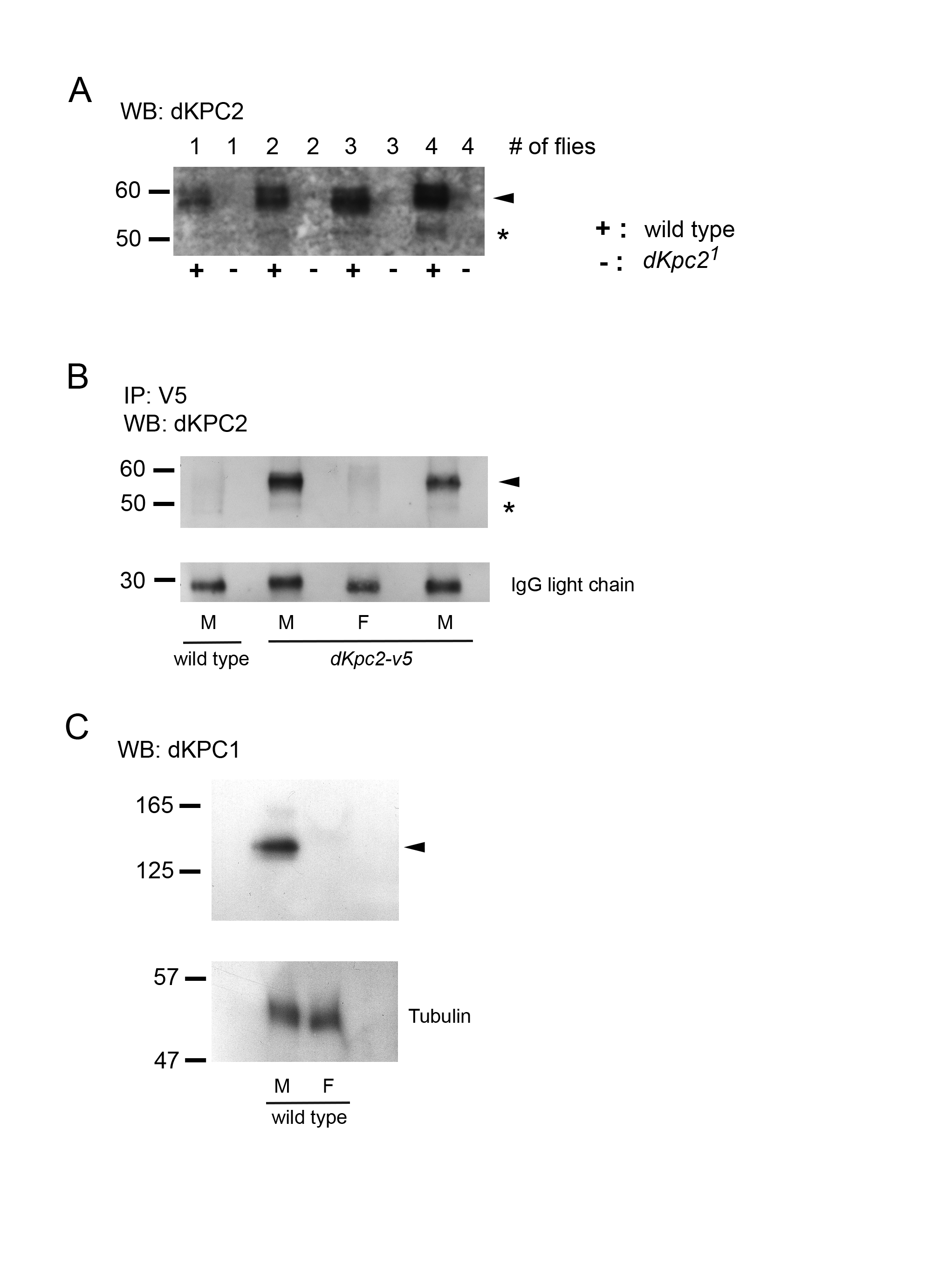

Supplement: S3 Fig — (A) Lysates were prepared from wild type and dKpc21 male flies as indicated. Western blots were probed with anti-dKPC2. dKPC2 is easily detected in as little as one wild type male fly but absent in dKpc21 males. (B) Extracts were prepared from male (M) or female (F) flies of the indicated genotypes and immunoprecipitated with mouse anti-V5 antibodies. Western blots were probed with anti-dKPC2 and goat anti-mouse IgG light chain as a loading control. dKPC2-V5 was only detected in male flies bearing the V5 tagged-dKpc2 genomic construct but not in wild type males or females having V5-dKpc2. (C) Lysates were prepared from wild type male (M) and female (F) flies. Western Blots were probed with anti-dKPC1. Loading control: Tubulin. dKPC1 protein is not detected in female flies. In panels A and B, the three dKPC2 isoforms are indicated by arrowheads and an asterisk. In panel C the arrowhead indicates the dKPC1 band. (TIF) [file pgen.1009217.s003.tif]

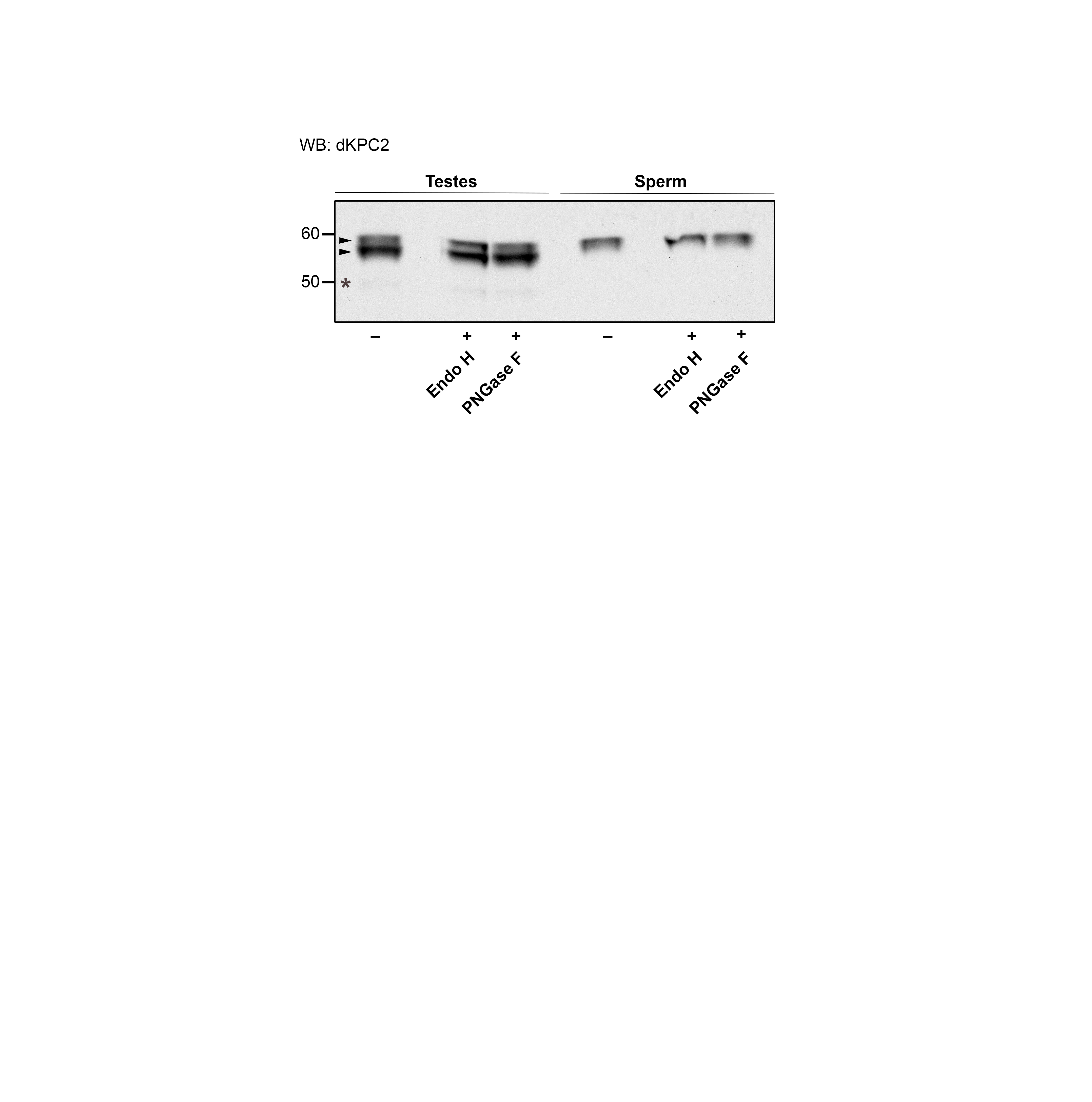

Supplement: S4 Fig — Total lysates from testis or sperm were treated with PNGaseF or EndoH as indicated. Western blots were probed with anti-dKPC2. dKPC2 resolves as 3 bands in testes but only the mature form is detected in sperm (see text) and there is no difference upon treatment of the lysates with either PNGaseF or EndoH. Black arrowheads indicate the dKPC2 doublet at ~58kDa. The asterisk denotes the unmodified product of Transcript B at ~49kDa. (TIF) [file pgen.1009217.s004.tif]

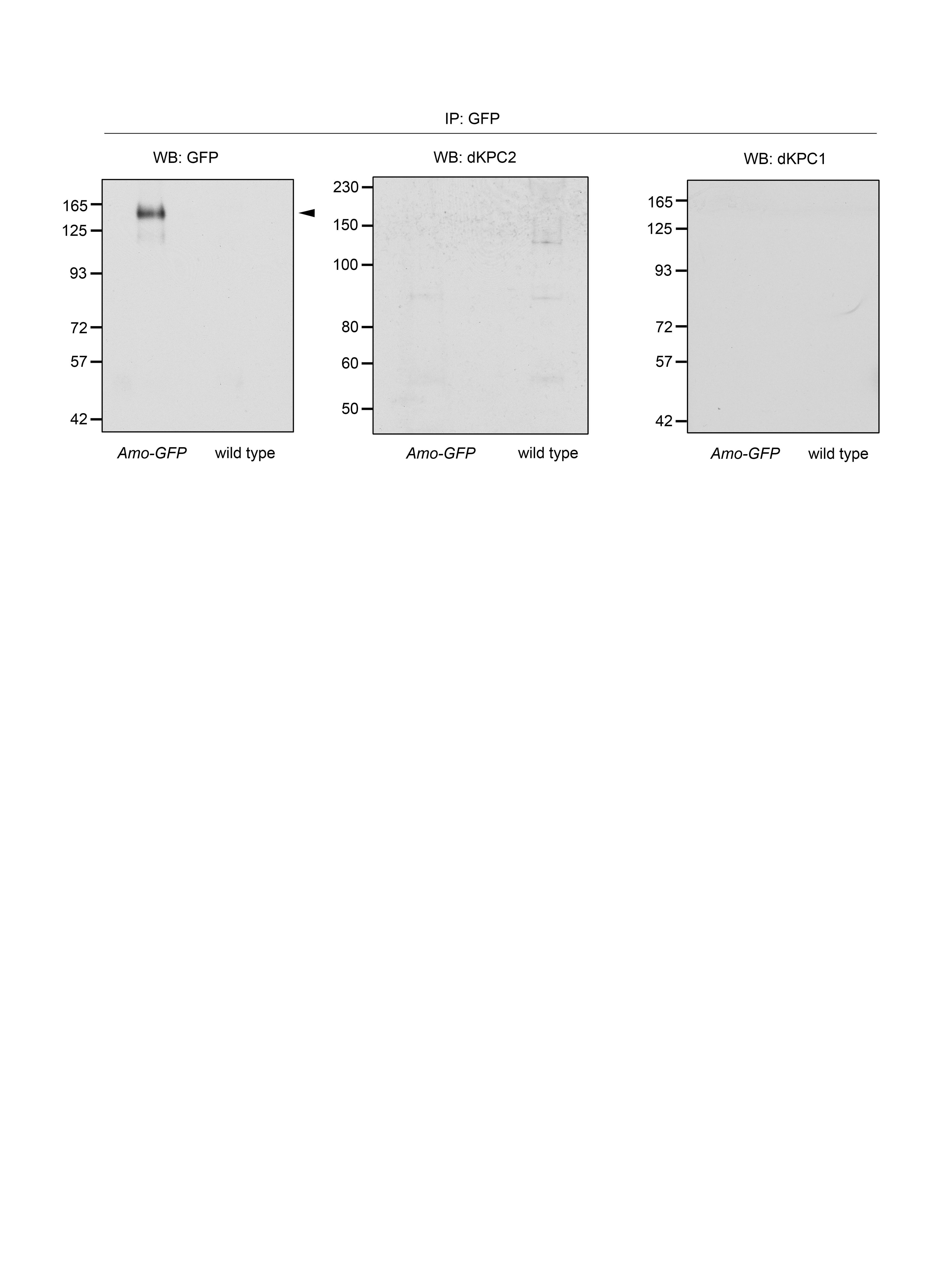

Supplement: S5 Fig — Extracts from Testes expressing Amo tagged at its C-terminus with GFP were immunoprecipitated with anti-GFP. Immune complexes were used to prepare Western blots that were probed with anti-GFP (left panel), anti-dKPC2 (middle panel) or anti-dKPC1 (right panel). Neither dKPC2 or dKPC1 co-IP with Amo. Amo-GFP is detected in the IP products. The Amo-GFP band, indicated by the arrowhead, is larger than the size of native protein (~105 kDa) due to the GFP tag. (TIF) [file pgen.1009217.s005.tif]

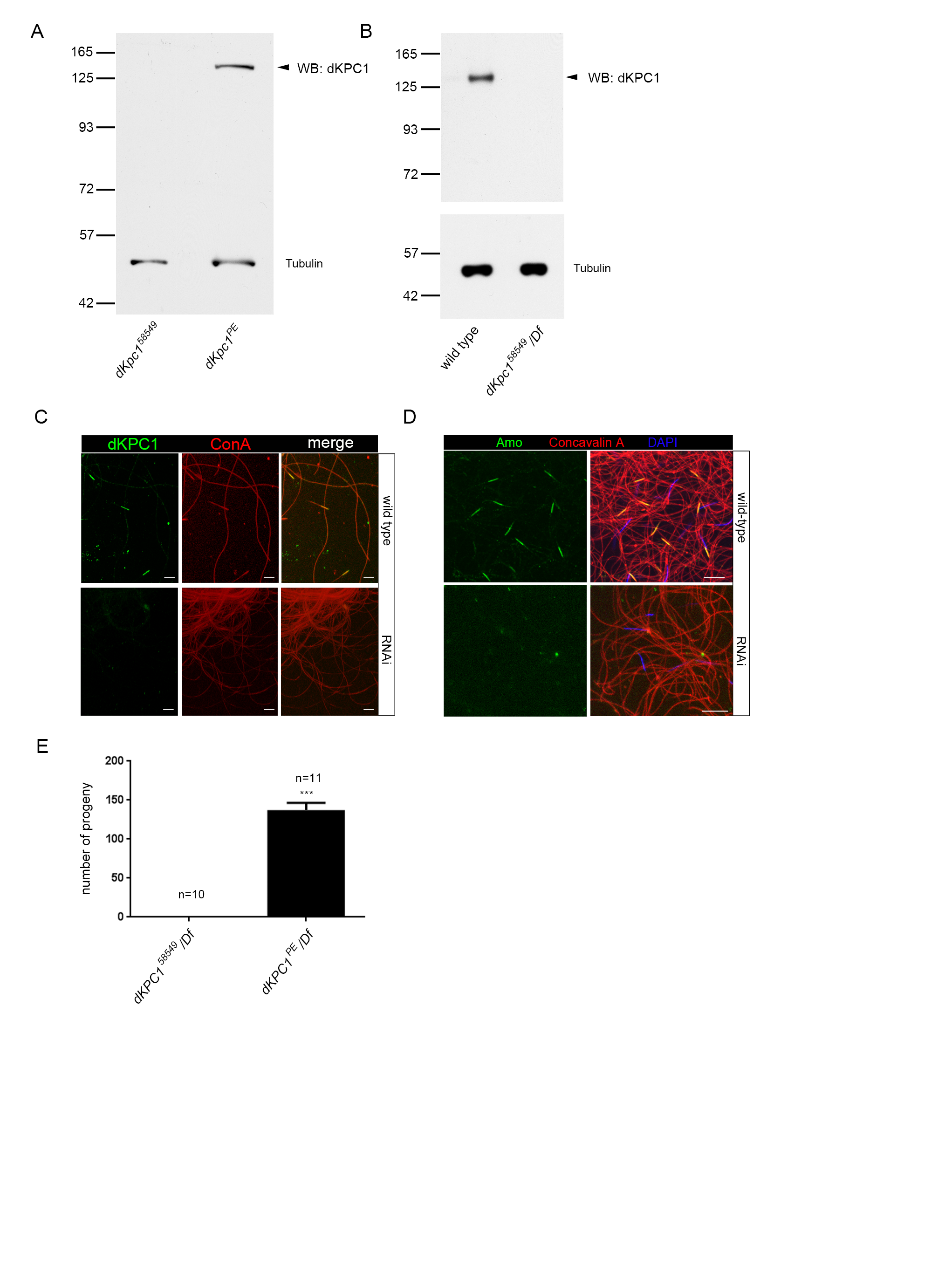

Supplement: S6 Fig — (A-B) Western blots of testes lysates probed with anti-dPKC1 (arrowhead). There is no detectable dKPC1 in flies with the MiMIC transposon insertion in dKpc1 (CG6752) exon 4 (dKpc158549) or in flies harboring the transposon in trans with a deletion (Df) that completely removes dKpc1. Precise excision of the transposon results in re-expression of dKPC1 (dKpc1PE). Tubulin serves as a loading control. (C) Sperm stained with anti-dKPC1: green, concanavalin A: red, DAPI: blue. dKPC1 staining is negative in sperm with RNAi mediated knock-down of dKPC1. Scale bars: 5 μm. (D) Sperm stained with anti-Amo: green, concanavalin A: red, DAPI: blue. Amo is missing from the sperm tail in flies with RNAi mediated knock-down of dKPC1. RNAi expression in the testis was driven by Bam-Gal4. Scale bars: 10 μm. (E) Fertility tests with males mated to wild type females. dKpc158549 males are sterile but fertility is rescued by precise excision of the transposon (dKpc1PE). The number of tests per genotype is denoted above the bars. *** P< 0.001. (TIF) [file pgen.1009217.s006.tif]

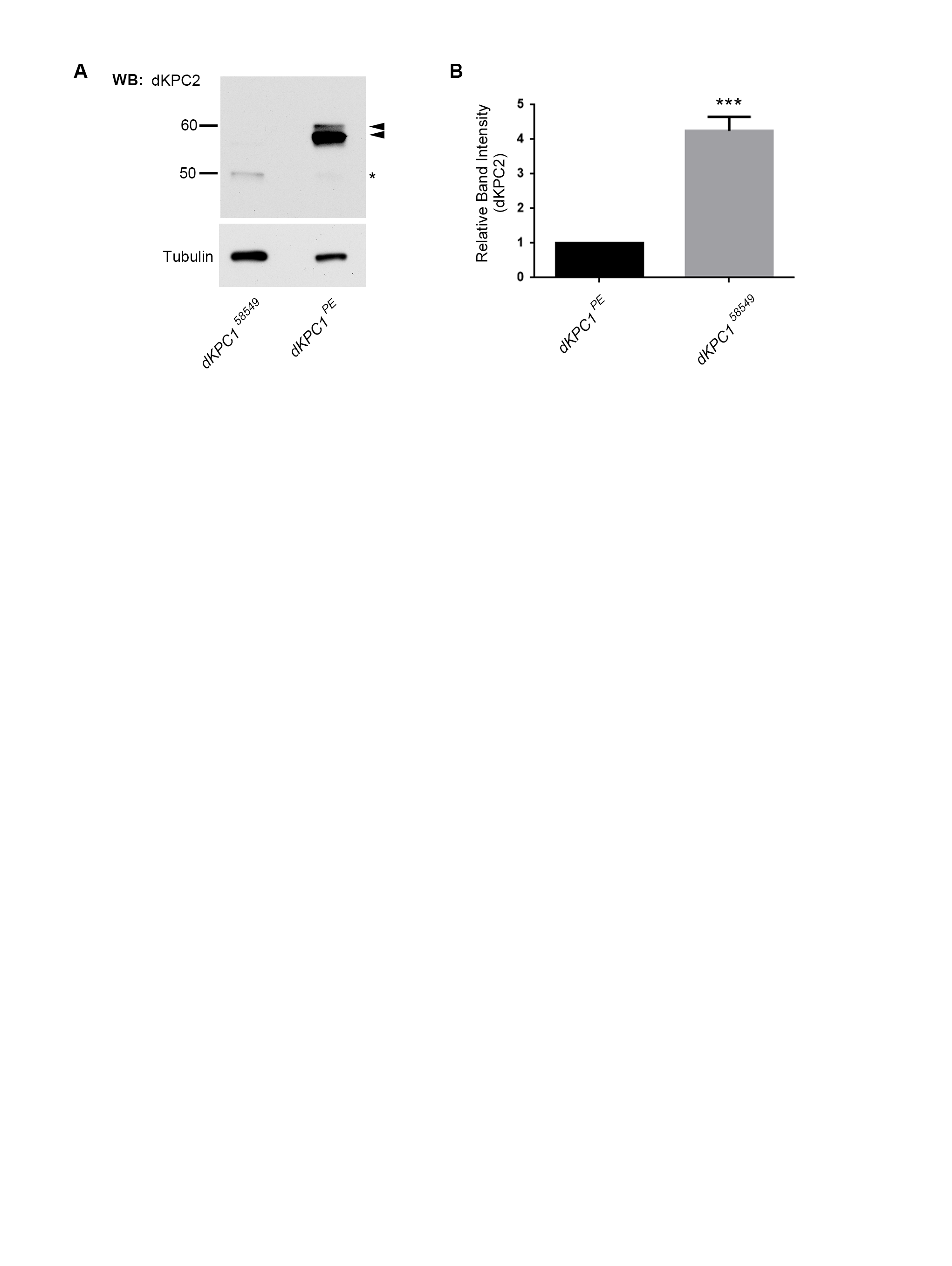

Supplement: S7 Fig — (A) Western blot prepared from testes lysates probed with Anti-dKPC2. The predominant dKPC2 isoform resolved in dKpc158549 testes is the ~49 kDa, unmodified protein, indicated by the asterisk). Precise excision of the transposon (dKpc1PE) yields testes expression of all three isoforms (arrowheads and asterisk) but the amount of the unmodified isoform (asterisk) appears to be reduced. Tubulin serves as a loading control. (B) Quantification of the ~49kDa dKPC2 isoform. The ratio of the dKPC2 ~49 kDa isoform of dKPC2 to Tubulin was calculated in dKpc158549 testes and normalized to dKpc1PE. The amount of unmodified dKPC2 in dKpc158549 mutant testes was ~4 times that in control. Graph shows quantification of 3 independent experiments. *** P< .001. (TIF) [file pgen.1009217.s007.tif]

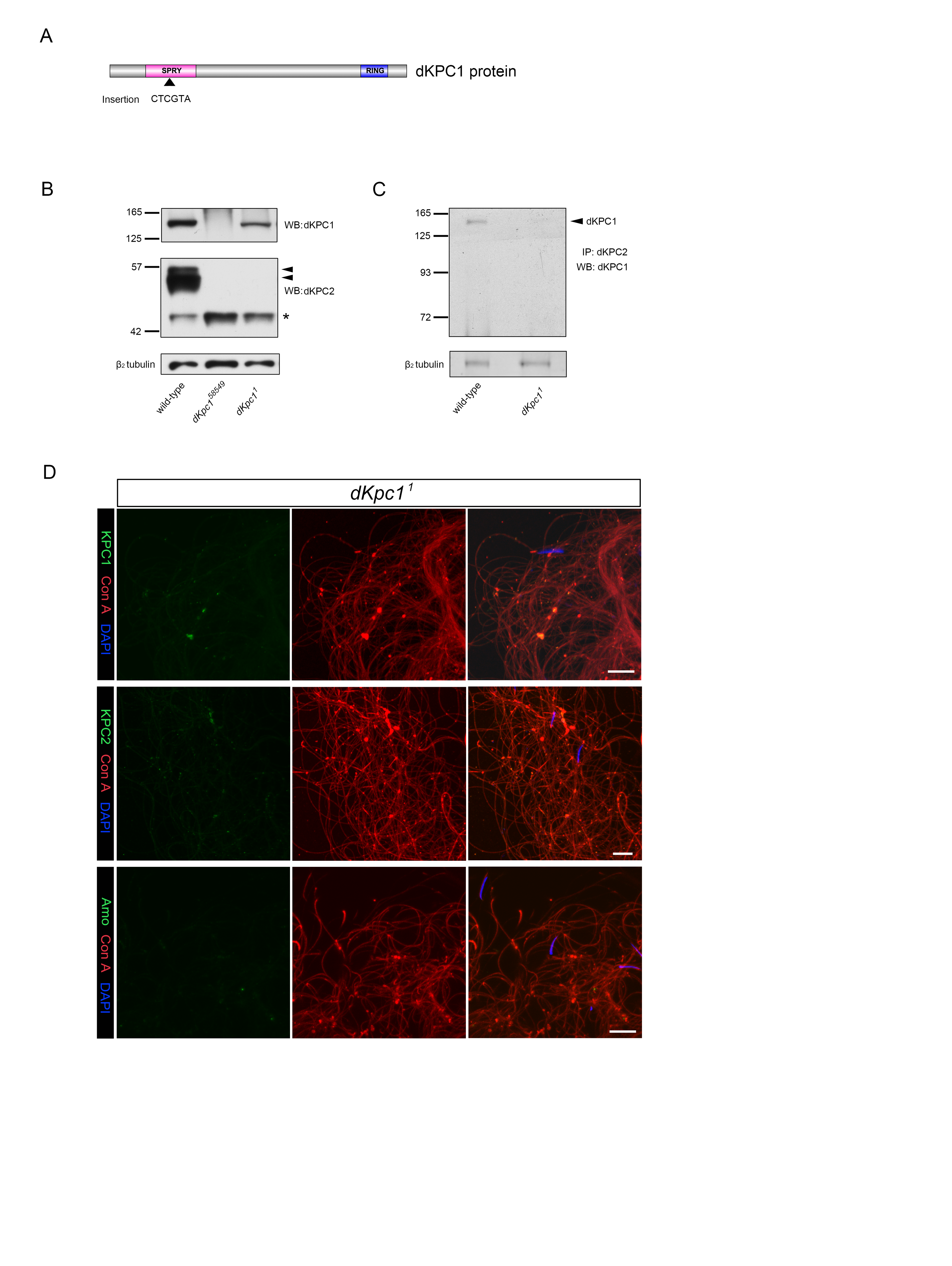

Supplement: S8 Fig — (A) Schematic of the dKpc11 allele, generated during transposase mediated excision, that contains a 6-base pair insertion in the predicted dKPC1 Spry domain. (B) Testes lysates were used to prepare Western blots that were probed with anti-dKPC1 (top panel) and anti-dKPC2 (middle panel). Tubulin serves as a loading control (lower panel). dKPC1 protein is still made in the dKpc11 allele but there are no ubiquitinated dKPC2 isoforms. Only the ~49 kDa isoform is present (asterisk). (C) Extracts from testes were immunoprecipitated with anti-dKPC2 and western blots were probed with anti-dKPC1 (arrowhead). dKPC2 and dKPC1 do not co-IP in the dKpc11 mutant, suggesting that ubiquitination is required for the interaction. (D) dKpc11 mutant sperm stained with anti-dKPC1 (green, top row), anti-dKPC2 (green, middle row) and anti-Amo (green, bottom row) along with concanavalin A: red and DAPI: blue. dKpc11 mutant sperm lack staining for all three proteins. Scale bars: 10 μm. (TIF) [file pgen.1009217.s008.tif]
